# Identification of Two Classes of Somatosensory Neurons That Display Resistance to Retrograde Infection by Rabies Virus

**DOI:** 10.1523/JNEUROSCI.1277-17.2017

**Published:** 2017-10-25

**Authors:** Gioele W. Albisetti, Alexander Ghanem, Edmund Foster, Karl-Klaus Conzelmann, Hanns Ulrich Zeilhofer, Hendrik Wildner

**Affiliations:** ^1^Institute of Pharmacology and Toxicology, University of Zurich, CH-8057 Zürich, Switzerland,; ^2^Max von Pettenkofer Institute & Gene Center, Virology, Faculty of Medicine, Ludwig-Maximilians-Universität München, 81377 München, Germany, and; ^3^Institute of Pharmaceutical Sciences, Swiss Federal Institute Zurich, CH-8090 Zürich, Switzerland

**Keywords:** neuronal circuits, rabies-mediated monosynaptic tracing, sensory system

## Abstract

Glycoprotein-deleted rabies virus-mediated monosynaptic tracing has become a standard method for neuronal circuit mapping, and is applied to virtually all parts of the rodent nervous system, including the spinal cord and primary sensory neurons. Here we identified two classes of unmyelinated sensory neurons (nonpeptidergic and C-fiber low-threshold mechanoreceptor neurons) resistant to direct and trans-synaptic infection from the spinal cord with rabies viruses that carry glycoproteins in their envelopes and that are routinely used for infection of CNS neurons (SAD-G and N2C-G). However, the same neurons were susceptible to infection with EnvA-pseudotyped rabies virus in tumor virus A receptor transgenic mice, indicating that resistance to retrograde infection was due to impaired virus adsorption rather than to deficits in subsequent steps of infection. These results demonstrate an important limitation of rabies virus-based retrograde tracing of sensory neurons in adult mice, and may help to better understand the molecular machinery required for rabies virus spread in the nervous system. In this study, mice of both sexes were used.

**SIGNIFICANCE STATEMENT** To understand the neuronal bases of behavior, it is important to identify the underlying neural circuitry. Rabies virus-based monosynaptic tracing has been used to identify neuronal circuits in various parts of the nervous system. This has included connections between peripheral sensory neurons and their spinal targets. These connections form the first synapse in the somatosensory pathway. Here we demonstrate that two classes of unmyelinated sensory neurons, which account for >40% of dorsal root ganglia neurons, display resistance to rabies infection. Our results are therefore critical for interpreting monosynaptic rabies-based tracing in the sensory system. In addition, identification of rabies-resistant neurons might provide a means for future studies addressing rabies pathobiology.

## Introduction

Rabies virus infection of different parts of the nervous system has been used for >20 years to identify functional networks of synaptically connected neurons (Ugolini, 1995, 2008). Unequivocal identification of monosynaptically connected neurons (monosynaptic retrograde tracing) has become possible through deletion of the glycoprotein (G-protein) gene from the genome of the rabies vaccine strain SAD B19 and inclusion of fluorescent reporter genes, such as GFP (Wickersham et al., 2007; for review, see Callaway and Luo, 2015; Ghanem and Conzelmann, 2016). Deletion of the G-protein coding sequence renders the virus noninfectious, while inclusion of a GFP expression cassette allows fast identification of infected neurons. Infectivity of the G-deleted rabies virus can be restored *in vivo* through trans-complementation of the virus with a G-protein expressed either from a plasmid, a helper virus, or a mouse transgene. This complementation enables monosynaptic retrograde spread and neurons providing direct synaptic input to the primary infected cells can then be identified by the expression of the reporter gene. This powerful approach has been rapidly adapted and is now widely used for circuit mapping throughout the nervous system (for review, see Callaway and Luo, 2015).

We and others have used this approach to identify sensory neuron subtypes providing input to defined types of second-order neurons in the spinal dorsal horn. The cell bodies of these sensory neurons are located in the dorsal root ganglia (DRG) from where they send axons to the periphery and to the spinal dorsal horn. Their peripheral terminals function as detectors of mechanical, thermal, proprioceptive, and noxious stimuli, while the central (spinal) terminals of these neurons form synaptic contacts with second-order neurons. A comprehensive characterization of the connections between DRG neurons and spinal neurons is crucial for our understanding of how different sensory modalities are processed, modified, and finally relayed to supraspinal sites where they are perceived.

Primary sensory nerve fibers can be subdivided broadly into myelinated (Aβ and Aδ) fibers and unmyelinated C-fibers. Myelinated Aβ fibers terminate predominantly in the deep dorsal horn (lamina III and deeper), while the terminals of unmyelinated C-fibers are found almost exclusively in laminae I and II. To identify the connections formed between sensory neurons and genetically defined spinal neuron populations, several groups of researchers have used monosynaptic rabies virus-based tracing methods (Bourane et al., 2015a,b; Foster et al., 2015; François et al., 2017; Sun et al., 2017). These reports provide strong evidence for direct synaptic input from myelinated sensory fibers. However, only one study has reported retrograde infection of unmyelinated nonpeptidergic [NP; equivalent to the isolectin B4 (IB4) binding population of sensory neurons] or tyrosine hydroxylase-positive unmyelinated C-fiber low-threshold mechanoreceptive (c-LTMR/TH+) sensory neurons. This is highly unexpected as IB4+ neurons account for ∼30% and TH+ for ∼14% of all sensory neurons innervating the lumbar spinal dorsal horn.

To systematically investigate the ability of G-deleted rabies virus to infect all classes of DRG neurons, we directly injected G-deleted rabies virus pseudotyped with SAD-G or N2C-G rabies glycoprotein into the spinal cord of wild-type mice and determined the identity of infected DRG neurons. Using a comprehensive set of markers, we found that TH+ c-LTMRs and unmyelinated NP DRG neurons were rarely infected. When initiating trans-synaptic tracing with SAD-G-complemented rabies virus from GRP-expressing spinal neurons, which are likely to receive c-LTMR and unmyelinated NP input, we found little infection of these subclasses of DRG neurons, suggesting that they display resistance to rabies-mediated retrograde infection. Using a recently developed optimized rabies G-protein (oG), we slightly increased the number of NP neurons that were infected, whereas almost no infection of TH+ neurons could be observed. Our results indicate that rabies-mediated tracing is biased toward myelinated neurons and is restricted from subsets of unmyelinated sensory neurons.

## Materials and Methods

### 

#### 

##### Mice.

Six-to-eight-week-old CB57BL/6 mice of either sex were used for the injections of SAD-G-pseudotyped and N2C-G-pseudotyped G-deleted rabies. Injections of SAD-G-pseudotyped G-deleted rabies were repeated in 129SVJ mice of either sex. SNS::Cre mice (Agarwal et al., 2004) and Grp::Cre mice (Gong et al., 2007) were crossed with Rosa26^lsl-TVA^ mice (Seidler et al., 2008) to enable infection with EnvA-pseudotyped rabies. SNS::Cre mice were also crossed with Rosa26^lsl-tdTomato^ mice (Ai14).

##### Immunohistochemistry and image analysis.

Mice were perfused with 4% paraformaldehyde (PFA) in PBS followed by postfixation in 4% PFA in PBS for 2 h. DRGs were cut into 16 μm cryosections and spinal cords were cut into 35 μm cryosections, which were mounted onto Superfrost Plus microscope slides (Thermo Fisher Scientific). All primary antibodies used in this study are listed in Table 2. Secondary cyanine 3-conjugated, Alexa Fluor 488-conjugated, DyLight 488-conjugated, DyLight 647-conjugated, and DyLight 649-conjugated donkey antibodies were obtained from Dianova. To detect IB4+ neurons, the isolectin IB4 Alexa Fluor 647 conjugate (Thermo Fisher Scientific) was used. Image analysis fluorescent images were acquired on a Zeiss LSM710 Pascal confocal microscope using a 0.8 numerical aperture ×20 Plan-apochromat objective or a 1.4 numerical aperture ×40 EC Plan-Neofluar oil-immersion objective and ZEN2012 software (Carl Zeiss). Whenever applicable, contrast, illumination, and false colors were adjusted in ImageJ or Adobe Photoshop (Adobe Systems). The number of immunoreactive cells was determined in 10 μm *z*-stacks using the ImageJ Cell Counter plug-in.

##### Rabies virus preparation and terminology.

All rabies viruses used in this study were derived from the SAD glycoprotein-deleted (ΔG) rabies in which the G-gene was replaced by either eGFP or mCherry (SAD.RabiesΔG.eGFP/mCherry). SAD.RabiesΔG.XFP viruses were pseudotyped with SAD-G, N2C-G, or EnvA glycoproteins, resulting in SAD.RabiesΔG.XFP (SAD-G), SAD.RabiesΔG.XFP (N2C-G), or SAD.RabiesΔG.XFP (EnvA) respectively. Pseudotyped SAD ΔG rabies virus was generated as described by Foster et al. (2015) and complemented as described previously (Wickersham et al., 2007). SAD.RabiesΔG.mCherry (N2c-G) was generated by transfecting HEK293T cells with pMD.RVG.CVS24-N2c (Addgene #19712, deposited by Manfred Schubert) and infecting the transfected cells with SAD.RabiesΔG.mCherry (SAD-G; multiplicity of infection, 0.01) Supernatants were harvested and concentrated as described previously (Wickersham et al., 2007).

##### Adeno-associated virus preparation.

AAV.flex.mCherry-2A-RabG vector was cloned in-house and packaged at the Penn Vector Core (Perelman School of Medicine, University of Pennsylvania) using their custom service. AAV.flex.mCherry-2A-RabG vector was cloned by excising the ChR2-mCherry fusion protein from pAAV-Ef1a-DIO-hChR2(H134R)-mCherryWPRE-pA (kindly provided by Dr. Karl Deisseroth, Stanford University) with AscI and NheI and replacing it with PCR amplified mCherry-2A-RabG cDNA. pAAV-Ef1a-DIO-oG-WPRE-hGH was obtained from Addgene (74290; Kim et al., 2016) and packaged by the Viral Vector Facility (Zurich). Adeno-associated virus (AAV) 2/1 vectors were used in this study.

##### Intraspinal virus injections.

Mice between 6 and 8 weeks old were anesthetized with 2–5% isofluorane and lumbar vertebrae L4 and L5 were exposed. Each animal was then placed in a motorized stereotaxic frame and the vertebral column was immobilized using a pair of spinal adaptors. The vertebral lamina and dorsal spinous process were removed to expose the L4 lumbar segment. The dura was perforated ∼500 μm to the left of the dorsal blood vessel using a beveled 30 ga needle. Viral vectors were injected at a depth of 200–300 μm using a glass micropipette (tip diameter, 30–40 μm) attached to a 10 μl Hamilton syringe. The rate of injection (30 nl/min) was controlled using a PHD Ultra syringe pump with a nanomite attachment (Harvard Apparatus). The micropipette was left in place for 5 min after the injection. In all experiments where rabies virus was injected, two individual injections at 500 nl each spaced ∼1 mm apart were made. Wounds were sutured and the animals were injected intraperitoneally with 0.03 mg/kg buprenorphine and allowed to recover on a heat mat. Rabies virus-injected mice were perfusion fixed 3–10 d after injection.

##### Retrograde tracing experiments.

For retrograde monosynaptic tracing experiments, we used a two-step strategy that involved injection of an AAV helper virus (AAV.flex.mCherry-2A-SAD-G or AAV.Ef1a.DIO.oG.WPRE.hGH) containing either a Cre-dependent mCherry and rabies glycoprotein (SAD-G) expression cassette or the cDNA of optimized rabies glycoprotein (oG) followed by a subsequent injection of an EnvA (avian sarcoma leukosis virus “A” envelop glycoprotein)-pseudotyped glycoprotein-deficient rabies virus [SAD.RabiesΔG.eGFP (EnvA)]. The tumor virus A (TVA) protein expressed from the Rosa26 reporter mouse line enabled cell type-specific infection of Grp::Cre+ neurons, and the SAD-G or oG was expressed to transcomplement the glycoprotein-deficient rabies virus in the primary infected neurons. Grp::Cre+ mice received two unilateral injections of AAV.flex.mCherry-2A-RabG/AAV.Ef1a.DIO.oG.WPRE.hGH [2.9 × 10^9^ genome copies (GC) per injection in 300 nl] into L3 and L4 segments of the dorsal horn. The vertebral lamina was left intact to limit the adhesion of scar tissue to the dorsal surface of the spinal cord. The mice were allowed to recover for 14 d and then received an injection of SAD.RabiesΔG.eGFP (EnvA; 1 × 10^6^ GC per injection in 500 nl) into the same site. Spinal cords and DRGs were harvested 5 d later.

##### Formalin injections.

For evaluation of the activity dependency of rabies infection, 10 μl of 5% formalin (Paraformaldehyde Granular, Electron Microscopy Science) dissolved in PBS was injected subcutaneously into the left hindpaw of the mouse immediately after the spinal injection of SAD.RabiesΔG.eGFP (SAD-G) virus. The mouse was kept under isoflurane anesthesia for 1 h following formalin injection. Buprenorphine (Tamgesic; 3 μg per mouse, s.c.) was given postoperatively before the mouse recovered from anesthesia.

##### Gene enrichment and pathway analysis.

To compare gene expression profiles of TH and PEP2, NF1, NF2 populations, we downloaded the external resource (Usoskin et al., 2015, their Table 2, http://linnarssonlab.org/drg/). In Excel, we applied the following two filter combinations: (1) ≤0.05 for the TH column and ≥0.2 for the PEP2, NF2, and NF3 columns; (2) ≤0.01 for the NF2, NF3, and PEP2 columns and ≥0.2 for the TH column.

The resulting gene lists were subjected to a pathway analysis using gene set enrichment analysis (GSEA) software and the Molecular Signature Database (Subramanian et al, 2005; http://www.broad.mit.edu/gsea/).

##### Experimental design.

For each experimental set, the respective pseudotyped rabies variant was injected into the dorsal horn of 4–8 mice. For all cell counts, sections were prepared from four to eight animals and ≥4 sections were analyzed per animal. To avoid the double counting of cells in adjacent sections, all sections used for quantification were taken ≥50 μm apart. To determine the prevalence of neurons in lumbar DRGs of CB57BL/6 wild-type mice, 6162 cells were evaluated. For the characterization of DRG neurons that became infected by spinal injection of SAD-G or N2C-G, 1059 and 1223 eGFP+ cells were analyzed, respectively. To determine the effect of neuronal activity on rabies transduction, 649 eGFP+ cells were analyzed. For the characterization of recombination-positive cells in SNS::Cre; Rosa26^lsl-tdTomato^ mice, 4820 cells were analyzed. To evaluate the ability of Enva-pseudotyped G-deleted rabies to transduce TVA-expressing Nav1.8+ neurons, 124 eGFP+ cells were analyzed. To determine the efficiency of trans-synaptic tracing initiated from spinal Grp+ neurons, 202 (SAD-G-complemented rabies) and 599 (oG-complemented rabies) eGFP+ cells were analyzed.

##### Statistics.

Microsoft Excel was used for statistical analysis. A two-way paired *t* test was used for the analysis of activity-dependent infection efficiencies.

## Results

### Prevalence of neuronal subsets in lumbar wild-type DRGs

**Figure 1.**
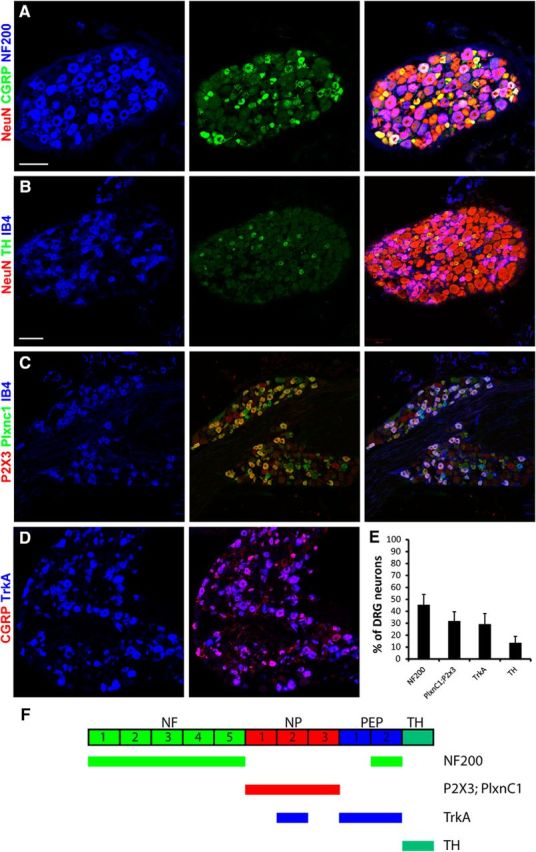
Characterization of the prevalence of neuronal subtypes in lumbar DRGs. ***A–D***, Immunohistochemical analysis of DRG neurons with antibodies against NeuN, CGRP, and NF200 (***A***); NeuN, TH, and IB4 (***B***); P2X3, PlxnC1, and IB4 (***C***); and CGRP and TrkA (***D***). ***E***, The relative abundance of DRG neurons immunoreactive for the indicated marker(s). ***F***, Schematic representation of which neuron classes are labeled by the antibodies or antibody combinations used in this study. Scale bar, 100 μm. Error bars represent the SD.

To understand whether rabies-mediated retrograde tracing allows infection of all sensory neuron populations, we analyzed their susceptibility to rabies virus infection in a systematic and comprehensive manner. We first evaluated suitable molecular markers for the identification of the different subclasses of DRG neurons that have recently been proposed (Usoskin et al., 2015). Based on single-cell RNA sequencing, this study suggested four major classes of DRG neurons: neurofilament-positive (NF+) neurons (including all LTMRs and proprioceptors), NP neurons (NP DRG neurons with unmyelinated axons), peptidergic (PEP) neurons (subdivided into unmyelinated PEP1 neurons and myelinated PEP2 neurons), and TH-expressing neurons (unmyelinated c-LTMRs). We compared molecular markers established by Usoskin et al., 2015 to the widely used IB4 labeling and CGRP (calcitonin gene-related peptide) antibody staining. We found that the IB4 binding population of sensory neurons largely overlaps with the Plxnc1;P2X3 double-positive population and that the TrkA+ and CGRP+ sensory neuron populations were virtually identical (Fig. 1*C*,*D*). A small fraction of Plxnc1;P2X3 double-labeled neurons did not bind IB4 and vice versa. When analyzing the fraction of NF200+ neurons, we found, as previously reported by others, that NF200+ neurons can be subdivided into NF200+;CGRP+ and NF200+;CGRP− neurons (Fig. 1*A*) and that the binding of IB4 and expression of TH are mutually exclusive (Fig. 1*B*). These findings are consistent with the results obtained by Usoskin at al. (2015). We therefore chose the following antibodies and antibody combinations for sensory neuron classification: anti-NF200 antibody (NF and PEP2 neurons), antibodies colabeled against Plxnc1 and P2X3 (NP neurons), antibodies against TrkA (PEP and NP2 neurons), and anti-TH antibodies (TH neurons/c-LTMRs; Fig. 1*F*). We then quantified the fraction of neurons labeled with these markers against the number of NeuN+ neurons in lumbar L3/L4/L5 DRGs of C57BL/6 wild-type mice. We found that 45.5 ± 8.5% of DRG neurons were NF200+ (corresponding to NF plus PEP2 neurons), 32.0 ± 7.7% were Plxnc1+;P2X3+ (NP neurons), 29.3 ± 8.7% were TrkA+ (NP2 and PEP), and 13.6 ± 5.4% were TH+ (TH neurons; Fig. 1*E*,*F*). These data agree with previously determined percentages of DRG neuron subtypes (McCoy et al., 2013).

### Injected SAD-G-pseudotyped or N2C-G-pseudotyped G-deleted rabies virus fails to infect NP and TH neurons

**Figure 2.**
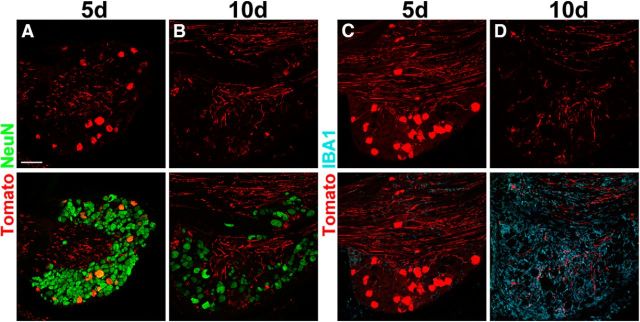
DRG neurons are sensitive to prolonged incubation with SAD ΔG rabies. ***A***, ***B***, Top row, mCherry (red) expression at 5 (***A***) and 10 d (***B***) after the injection of SAD.RabiesΔG-mCherry (SAD-G). Bottom row, colabeling of mCherry and NeuN is depicted (green). ***C***, ***D***, Immunohistochemical analysis of SAD.RabiesΔG-mCherry-infected DRGs with antibodies against mCherry (red) and the macrophage marker IBA1 (cyan) at 5 and 10 d after spinal injection. mCherry expression is depicted in the top row; colabeling of mCherry and IBA1 is depicted in the bottom row. Note the reduced number of mCherry+ cell bodies at 10 d after injection and the strong upregulation of the macrophage marker IBA1. SAD.RabiesΔG-mCherry (SAD-G) was injected intraspinally at 2.4 × 10^8^ focus forming units per milliliter. Scale bar, 100 μm.

SAD-G rabies glycoprotein has been used for transcomplementation in most monosynaptic retrograde tracing studies. To determine whether rabies-G-coated modified rabies can equally infect the central terminals of primary afferents of all classes of DRG neurons, we injected SAD.RabiesΔG-mCherry (SAD-G) or SAD.RabiesΔG-eGFP (SAD-G) into the spinal cord of C57BL/6 wild-type mice. We first determined an optimal incubation time for our analysis. A longer incubation time will lead to increased fluorescence due to an accumulation of the rabies-encoded marker gene product, but infection with modified rabies is cytotoxic after a certain incubation time (Wickersham et al., 2007; Reardon et al., 2016). In our hands, DRG neurons are sensitive to rabies infection and start to downregulate endogenous proteins, such as NeuN at 7–8 d after infection. However, other groups have found maximum infection efficiency after 10 d in different parts of the nervous system (Wertz et al., 2015). We therefore analyzed infection of DRG neurons at 5 and 10 d following intraspinal injection of rabies virus. Five days after intraspinal injection of SAD.RabiesΔG-mCherry (SAD-G), all infected DRG neurons expressed NeuN (Fig. 2*A*). In contrast, at 10 d after injection, we rarely found any mCherry+ DRG cell bodies, although mCherry+ axons were still detectable (Fig. 2*B*). In addition, we observed a strong increase in macrophage infiltration evident by the upregulation of IBA1 at 10 d compared with 5 d after injection (Fig. 2*C*,*D*). This suggests that prolonged expression and replication of the SAD.RabiesΔG-mCherry genome rapidly becomes cytotoxic for DRG neurons. We therefore performed subsequent experiments 5 d after injection.

**Figure 3.**
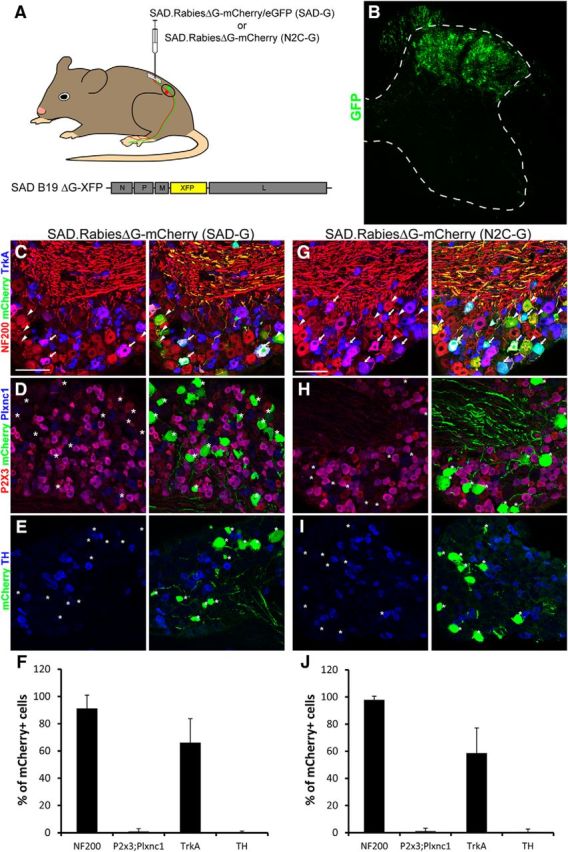
Molecular profiling of SAD.RabiesΔG-eGFP (SAD-G)-infected and SAD.RabiesΔG-mCherry (N2C-G)-infected DRG neurons. ***A***, Schematic representation of the experimental design. SAD-G-pseudotyped RabiesΔG-mCherry/eGFP or N2C-G-pseudotyped RabiesΔG-mCherry was injected at L3–L5 levels of the lumbar spinal cord of wild-type mice. mCherry/eGFP expression from the rabies genome enables tracing of infected neurons. ***B***, eGFP expression in a representative image of a lumbar spinal cord section 5 d after infection. Note that infection of local interneurons predominantly occurs in laminae I–IV. The dotted white line outlines the spinal gray matter. ***C–E***, ***G–I***, Immunohistochemical analysis of infected DRG neurons with antibodies against NF200, mCherry, and TrkA (***C***, ***G***), with antibodies against P2X3, mCherry, and Plxnc1 (***D***, ***H***), and with antibodies against mCherry and TH (***E***, ***I***). Left column in ***C–E*** and ***G–I*** shows expression of the indicated markers and the right column depicts the indicated markers together with the rabies-derived mCherry. Arrows indicate mCherry+ neurons labeled with two additional markers. Arrowheads indicate mCherry+ neurons labeled with one additional marker. Asterisks indicate mCherry+ neurons negative for the analyzed marker. ***F***, ***J***, Quantification of mCherry+ neurons characterized by the expression of the respective marker after spinal injection of either SAD.RabiesΔG-mCherry (SAD-G; ***F***) or SAD.RabiesΔG-mCherry (N2C-G; ***J***). SAD.RabiesΔG-mCherry (SAD-G) and SAD.RabiesΔG-mCherry (N2C-G) were adjusted to the same titer and subsequently injected at 2.4 × 10^8^ focus forming units per milliliter. Scale bars, 100 μm. Error bars represent the SD.

Five days after injection of SAD.RabiesΔG-mCherry/eGFP (SAD-G) into the left lumbar dorsal horn, numerous neuronal cell bodies throughout laminae I–IV became positive for the respective fluorescent marker (Fig. 3*A*,*B*), suggesting that the primary afferent terminals of this area were also exposed to the rabies virus. We then analyzed the molecular profile of SAD.RabiesΔG-mCherry-infected (mCherry+) DRG neurons and found that 91.3 ± 9.7% were NF200+ (NF plus PEP2 neurons), 1 ± 2% were Plxnc1+;P2X3+ (NP), 66.2 ± 17.5% were TrkA+ (NP2, PEP), and 0.2 ± 1% were TH+ (TH; Fig. 3*C–F*). Compared with the prevalence of neurons labeled with each respective marker in wild-type DRGs (Fig. 1*E*), Plxnc1+;P2X3+ and TH+-labeled neurons were under-represented whereas NF200+-labeled and TrkA+-labeled neurons were over-represented. Almost identical results were obtained when using SAD.RabiesΔG-eGFP (SAD-G) or a different genetic background of mice (129SvJ).

To exclude the possibility that the inability of SAD.RabiesΔG-mCherry (SAD-G) to infect NP or TH neurons was specific to the SAD-G-protein derived from an attenuated vaccine strain, we repeated these experiments with SAD.RabiesΔG-mCherry (N2C-G), which was pseudotyped with the G-protein of the challenge virus strain variant N2C (N2C-G; Fig. 3*A*). This strain has been reported to have higher neurotropism (Morimoto et al., 1998; Reardon et al., 2016). As in the previous experiment, we observed neuronal loss at 10 d but not at 5 d after injection of SAD.RabiesΔG-mCherry (N2C-G; data not shown). When we determined the neuronal identity 5 d after injection, we obtained results very similar to those obtained after injection of SAD.RabiesΔG-mCherry (SAD-G). Of the mCherry-labeled DRG neurons, 97 ± 2.6% were NF200+, 1 ± 2.2% were Plxnc1+;P2X3+, 58 ± 18.8% were TrkA+, and 0.7 ± 2.1% were TH+ (Fig. 3*G–J*), indicating that the central terminals of NP and TH neurons are largely resistant to retrograde infection with either SAD-G-pseudotyped or N2C-G-pseudotyped rabies virus.

**Figure 4.**
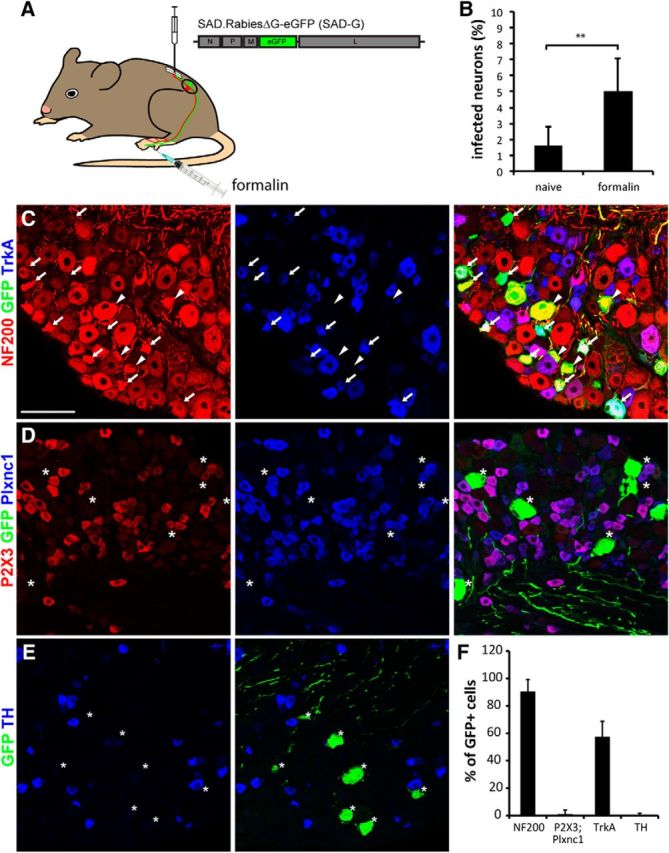
Evaluation of the activity dependency of rabies virus infection. ***A***, Schematic representation of the experimental design. SAD-G-pseudotyped rabiesΔG-eGFP was injected at L3–L5 levels of the lumbar spinal cord of wild-type mice followed immediately by the injection of formalin into the hindpaw that provides innervation to the spinal injection site. ***B***, Quantification of the percentage of infected DRG neurons in naive and formalin-injected mice. ***C–E***, Immunhistological stainings with antibodies against GFP, NF200, and TrkA (***C***); GFP, P2X3, and Plxnc1 (***D***); and GFP and TH (***E***). ***F***, Quantification of the percentage of infected neurons expressing the indicated marker (**p < 0.01). SAD.RabiesΔG-eGFP (SAD-G) was injected at 4.5 × 10^7^ focus forming units per milliliter. Scale bars, 100 μm. Error bars represent the SD.

We next addressed whether the bias toward infection of myelinated sensory neurons was due to different activity levels of sensory neuron subtypes during exposure to the rabies virus. In unchallenged mice, myelinated touch-sensitive neurons and proprioceptive neurons likely exhibit higher levels of activity than unmyelinated nociceptors and pruritoceptors. To test whether neuronal activity increases rabies virus infection, we injected the rabies virus into the spinal cords of mice under conditions that tonically activate nociceptors. We injected the TRPA1 agonist formalin (McNamara et al., 2007) subcutaneously into the hindpaw of mice immediately after spinal injection of SAD.RabiesΔG-eGFP (SAD-G; Fig. 4*A*). Subcutaneous injection of formalin induces tonic activity in nociceptive nerve fibers that innervate the injected skin area (Fischer et al., 2015). The IB4+ subpopulation of unmyelinated sensory neurons (equivalent to NP neurons), most of which function as nociceptors or pruritoceptors, is highly susceptible to formalin excitation as expression of TRPA1 channels is particularly strong in these neurons (Barabas et al., 2012). When comparing the infection rate of sensory neurons between naive mice and formalin-treated mice, we found that there was indeed a significant increase in the percentage of labeled DRG neurons in the formalin-injected group (5.0 ± 2.1% in formalin-injected mice vs 1.6 ± 1.2% of labeled neurons in naive mice, *t* test *p* = 0.007; Fig. 4*B*). However, despite this formalin-induced increase in the total number of infected DRG neurons, we still did not find infected NP or TH neurons (Fig. 4*C–F*). The resistance of these neurons to infection by SAD.RabiesΔG-eGFP (SAD-G) is hence unrelated to low neuronal activity during virus exposure.

### NP neurons can support virus replication and expression of viral genes

**Figure 5.**
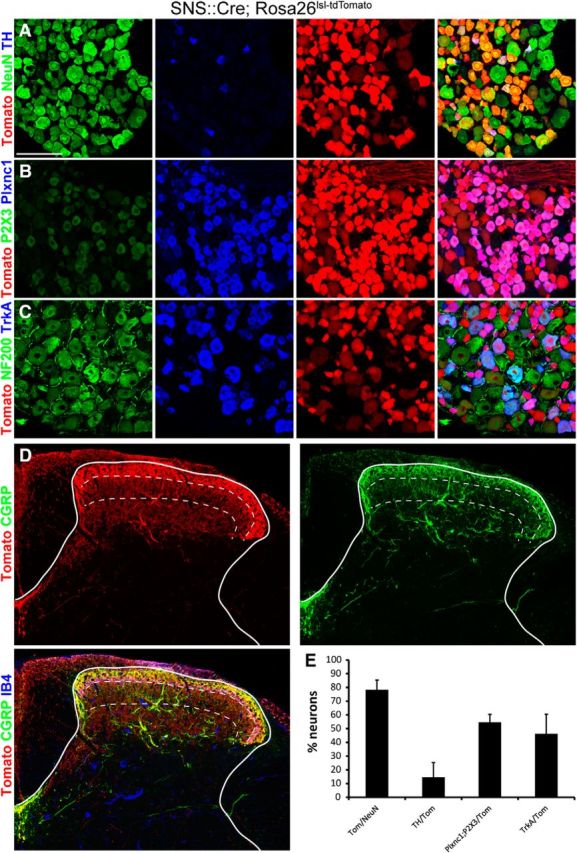
Characterization of the molecular identity of DRG-expressing Cre in SNS::Cre mice. ***A–C***, Immunohistochemical analysis of lumbar DRG neurons in SNS::Cre; Rosa26^lsl-Tomato^ mice. ***A***, Analysis of Tomato+ DRG neurons with antibodies against NeuN and TH. ***B***, Analysis of Tomato+ DRG neurons with antibodies against P2X3 and Plxnc1. ***C***, Analysis of Tomato+ DRG neurons with antibodies against NF200 and TrkA. ***D***, Termination zone of Tomato+ primary afferent fibers in the spinal cord. Dotted lines show the borders between LII and LIII and between LIII and deeper dorsal horn. ***E***, Quantification of the proportion of Tomato+ DRG neurons of all DRG neurons (Tom/NeuN) and quantification of the percentage of the indicated markers among all Tomato+ neurons. Scale bar, 100 μm. Error bars represent the SD.

**Figure 6.**
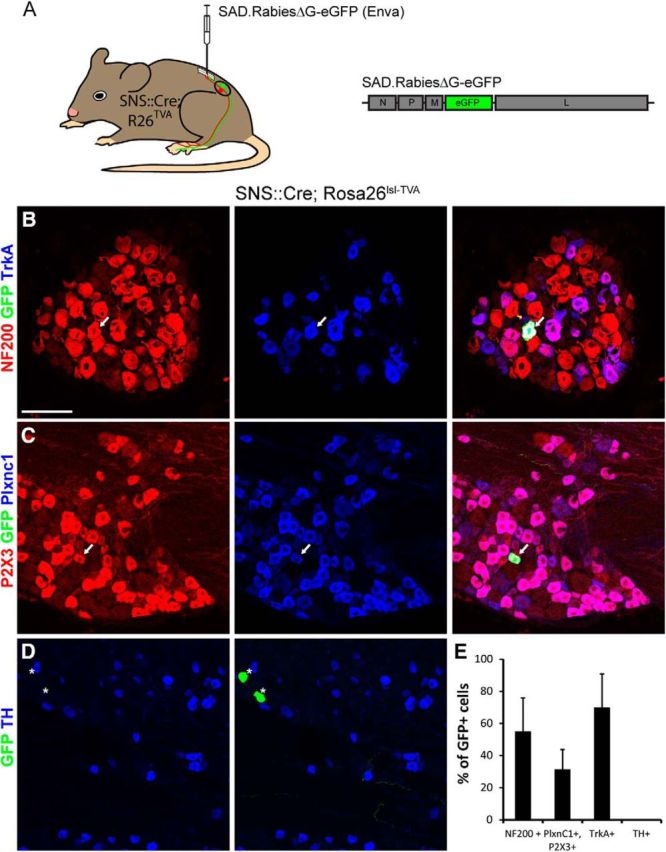
Molecular profiling of SAD.RabiesΔG-eGFP (Enva)-infected DRG neurons from SNS::Cre; Rosa26^lsl-TVA^ mice. ***A***, Schematic representation of the experimental design. SAD.RabiesΔG-eGFP (Enva) contains the same genome as SAD.RabiesΔG-eGFP (SAD-G), but is pseudotyped with the EnvA glycoprotein instead. SAD.RabiesΔG-eGFP (Enva) was injected into L3–L4 levels of the lumbar spinal cord of SNS::Cre; Rosa26^lsl-TVA^ mice. ***B***, Characterization of eGFP+ neurons with antibodies directed against NF200 and TrkA. ***C***, Detection of a P2X3+;Plxnc1+-infected DRG neuron (eGFP+). ***D***, Analysis of infected DRGs with antibodies against GFP and TH. Arrows indicate eGFP+ neurons labeled with two additional markers. Asterisks indicate eGFP+ neurons negative for the analyzed marker. ***E***, Quantification of the percentage of eGFP+ neurons expressing the indicated marker. SAD.RabiesΔG-eGFP (Enva) was injected at 3.5 × 10^8^ focus forming units per milliliter. Scale bars, 100 μm. Error bars represent the SD.

We then tested whether the absence of efficient labeling of TH or NP neurons was due to the inability of rabies G-coated SAD.RabiesΔG-eGFP to enter these neurons or whether it was due to a deficiency in some postinfection processes, such as the axonal transport of the SAD.RabiesΔG-eGFP, viral transcription and replication, or translation of the proteins encoded by the rabies virus genome. To determine the answer, we first generated sns::Cre; Rosa26^lsl-TVA^ double-transgenic mice, in which the Cre-recombinase is expressed in all unmyelinated sensory neurons (Agarwal et al., 2004). We then injected the lumbar spinal cord of these double-transgenic mice with EnvA-pseudotyped SAD.RabiesΔG-eGFP. This strategy bypasses the typical pathway of virus entry and direct infection of unmyelinated fibers via the interaction of the EnvA G-protein with the TVA receptor. To verify that NP and TH neurons of sns::Cre; Rosa26^lsl-TVA^ mice expressed the TVA receptor, we first characterized the recombination pattern mediated by the sns::Cre driver in sns::Cre; Rosa26^lsl-Tomato^ mice. In these mice, Plxnc1+;P2X3+, TrkA+, and all TH+ neurons expressed Tomato, indicating that Cre was also expressed in all NP, PEP, and TH neurons (Fig. 5*A–C*). In the spinal cord, strong Tomato+ central axons were seen in LI and LII but weaker expression was also observed in LIII and LIV (Fig. 5*D*). As expected, Tomato− DRG neurons were NF200+ but TrkA− and thus belonged to the NF population of non-nociceptive somatosensory and proprioceptive neurons (Fig. 5*C*). Conversely, we found that 54.5% of Tomato+ neurons were Plxnc1+;P2X3+, 46.3% were TrkA+, and 14.6% were TH+ (Fig. 5*E*). We then injected SAD.RabiesΔG-eGFP (EnvA) into the spinal cord of sns::Cre; Rosa26^lsl-TVA^ mice (Fig. 6*A*) and characterized the markers expressed by the eGFP+ DRG neurons. Fifty-nine percent of the eGFP+ neurons expressed NF200, 38% coexpressed Plxnc1 and P2X3, and 68% expressed TrkA, but we found no coexpression with TH (Fig. 6*B–E*). Expression of eGFP in Plxnc1+;P2X3+ neurons indicated that NP neurons possess the machinery to support virus replication and the expression of proteins encoded by the rabies virus genome. It is therefore likely that the failure to infect the central terminals of NP neurons underlies the resistance of NP DRG neurons to SAD-G/N2C-G-coated, rabies-mediated infection.

### TH and NP neurons are resistant to trans-synaptic tracing with SAD-G-pseudotyped rabies, but display low susceptibility to trans-synaptic tracing with oG-pseudotyped rabies

Ruigrok et al. (2016) recently suggested that infection via the extracellular space and transneuronal/trans-synaptic infection might rely on different mechanisms. To test whether NP and TH neurons are equally resistant to either transneuronal or direct spinal infection by G-complemented rabies, we investigated a spinal subpopulation that should receive input from NP and TH neurons.

**Figure 7.**
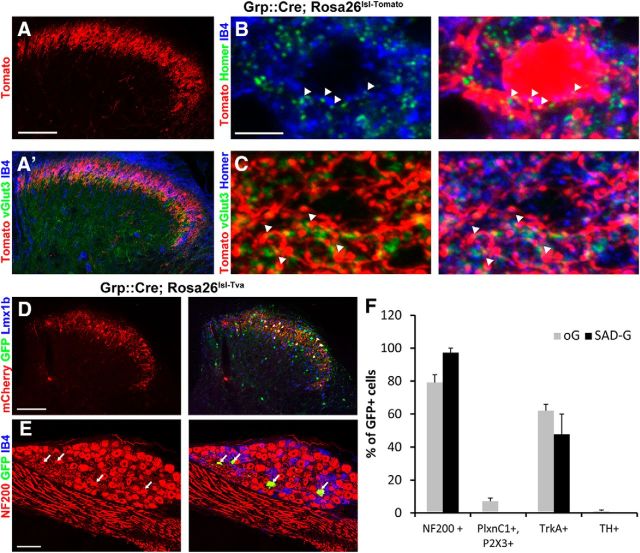
Monosynaptic tracing initiated from spinal Grp::Cre neurons. ***A***, ***A'***, Grp::Cre neurons are located in lamina II of the spinal cord. The neuropil and cell bodies of GRP::Cre neurons overlap with central terminals of IB4+ and TH+ primary afferent fibers. ***B***, High-resolution imaging of a tdTomato+ Grp neuron (red) indicates the presence of Homer1+ (green) postsynapses on Grp neurons, which are near IB4+ (blue) terminals. ***C***, High-resolution imaging of tdTomato+ Grp neuropil (red) indicates the presence of Homer1+ (blue) postsynapses on Grp neurons near vGlut3+ (green) primary afferent terminals. ***D***, Rabies-mediated monosynaptic retrograde tracing reveals many primary (mCherry+ and eGFP+) and secondary (only eGFP+) infected spinal neurons. ***E***, Secondary infected DRG neurons are mostly NF200+ (arrows). ***F***, Quantification of eGFP-labeled DRG neuron subtypes after retrograde transduction mediated by either SAD-G or oG rabies glycoprotein. SAD.RabiesΔG-eGFP (Enva) was always injected at 3.5 × 10^8^ focus forming units per milliliter. AAV1.EF1a.flex.mCherry-2A-SADG and pAAV1.Ef1a.DIO.oG.WPRE.hGH were injected at 9.5 × 10^12^ GC/ml. Scale bars: ***A***, ***D***, ***E***, 100 μm; ***B***, ***C***, 5 μm. Error bars represent the SD.

Spinal Grp+ neurons are second-order excitatory neurons located in an area of the superficial dorsal horn that overlaps with the termination zone of NP and TH neurons (LIIi and LIIiv respectively). In sections obtained from Grp::Cre; Rosa26^lsl-Tom^ mice, a profound regional overlap of tdTomato with CGRP+ (PEP and NP2), IB4+ (NP), and vGlut3+ (TH) immunofluorescence was observed (Fig. 7*A*). To provide further evidence for direct synaptic contacts between Grp neurons and IB4+ and/or vGlut3+ primary sensory axon terminals, we used IB4/vGlut3 labeling in combination with an antibody against the postsynaptic marker protein Homer1 on Grp::Cre; Rosa26^lsl-Tom^ mice (Gutierrez-Mecinas et al., 2016). We found IB4+ and vGlut3+ terminals directly opposing Homer1-labeled postsynapses on Grp neurons (Fig. 7*B*,*C*).

To initiate retrograde tracing from Grp+ spinal neurons, Grp::Cre mice were crossed with TVA reporter mice (Rosa26^lsl-Tva^). Double-transgenic mice derived from these crosses were injected with an AAV.flex.mCherryT2A-SAD.G helper virus into the left lumbar spinal cord to allow complementation of the G-deleted rabies in Cre+ neurons. Two weeks later, we injected pseudotyped SAD.RabiesΔG-eGFP (EnvA) into the same site. Five days after rabies virus injection, we observed numerous eGFP+ neurons in the spinal cord. Many of these were mCherry−, suggesting successful secondary (retrograde) infection (Fig. 7*D*). We also detected numerous eGFP+ neurons in the DRGs of these injected mice (Fig. 7*E*). None of the DRG neurons were mCherry+, verifying that these neurons were secondary infected cells. We then analyzed the molecular profile of the retrogradely infected DRG neurons. Using the same set of antibodies as before, we found that nearly all infected neurons (97 ± 2.7%) were NF200+. Approximately half of these neurons also expressed TrkA (47.8 ± 12.1%), but none of the infected neurons were found to be NP or TH neurons (Fig. 7*F*).

Recently an optimized rabies G-protein (oG) has been developed to allow for more efficient trans-synaptic tracing (Kim et al., 2016). We therefore repeated the trans-synaptic-tracing experiment and injected an AAV.flex.oG helper virus into the spinal cord of Grp::Cre; Rosa26^lsl-Tva^. Indeed, when analyzing the identity of infected DRG neurons, we found a shift in the molecular profile. Use of the oG rabies G-protein led to a relative increase in infected TrkA+ versus NF200+ DRG neurons. In these experiments, a small number of infected NP (7.2 ± 2%) and TH neurons (1.2 ± 0.7%) was also apparent (Fig. 7*F*). However, given the abundance of TH and IB4 terminals forming synaptic contacts with Grp neurons, the number of infected NP neurons, and especially TH neurons, was much lower than expected. This indicated a high degree of resistance of TH and IB4 neurons to rabies virus infection, including those pseudotyped with the oG glycoprotein.

### TH neurons show differences in expression of membrane proteins compared with DRG subpopulation highly susceptible to rabies infection

We have identified DRG neurons of the TH class as being largely resistant to SAD ΔG rabies virus pseudotyped with three different variants of rabies glycoprotein. We reasoned that the resistance to rabies infection displayed by TH neurons could either be due to the lack of proteins required for rabies uptake, transport, or unloading and translation of the rabies genome or to the presence of proteins that prevent any of these steps. To obtain first insights into the potential mechanisms conferring resistance to TH neurons, we analyzed whole transcriptome data provided by Usoskin and colleagues (Usoskin et al., 2015, their Table 2; http://linnarssonlab.org/drg/). First, we analyzed these data for the expression of three protein families that have been described as potential rabies receptors. These included the nicotinic acetylcholine receptor (nAChR), the neural cell adhesion molecule (NCAM), and the low-affinity neurotrophin receptor (p75NTR/Ngfr; Lentz et al., 1983; Thoulouze et al., 1998; Tuffereau et al., 1998a,b; Langevin and Tuffereau, 2002). NCAM1/2 and p75NTR (Ngfr) were expressed in a similar or even higher proportion of TH cells when compared with the NF and PEP2 subclasses, which were susceptible to rabies infection (Table 1). Also among the different nAChR subunits, we could not identify any that were expressed in NF and PEP2 neurons but absent from TH neurons (Table 1). In our analysis, we found rabies infected NF200+ DRG neurons that belonged to the PEP2 (NF200+;TrkA+) and NF2/3 subgroups (NF200+;Calb1+ or NF200+;TrkC+; data not shown; markers for NF1 or NF4/5 were not analyzed). We next performed a genome-wide analysis and exploited again the transcriptomics data from Usoskin et al. (2015) and searched for genes that were either enriched or depleted in TH neurons. To this end, we applied two criteria. First, we filtered for genes detected in ≥20% of the susceptible subpopulations but in <5% of the TH class. Second, we identified all genes that were detected in ≥20% of the TH population but in <10% of NF2/3 and PEP populations. We identified 321 genes not expressed in TH neurons and 52 genes detected in >20% of TH neurons but not in PEP2 and NF2/3 neurons (see Table 1-1 and Table 1-2). GO-term pathway analysis (GSEA) of the list of genes lacking in TH neurons revealed that the functions of the differentially expressed genes relate to “neuron part synapse,” “neuron projection,” “regulation of anatomical structure morphogenesis,” and “regulation of transport.” These results suggest that resistant TH neurons and sensitive PEP2 and NF2/3 neurons differ significantly in synapse function or morphology. As wild-type rabies virus spreads in the CNS via synapses, it is tempting to speculate that some of these genes are involved in neuronal infection by rabies virus.

**Table 1.** Expression of known rabies receptors in DRG subpopulations

Gene	NF					NP			PEP		TH
	NF1	NF2	NF3	NF4	NF5	NP1	NP2	NP3	PEP1	PEP2	
*Ncam1*	6.5	0	0	4.5	0	32.8	31.3	16.7	28.1	11.7	55.8
*Ncam2*	13	8.3	25	0	3.8	1.6	3.1	0	7.8	41.2	1.3
*Chrna1*	0	6.3	33.3	0	0	0	0	0	0	0	0.4
*Chrna10*	0	0	8.3	0	0	0	0	0	0	17.6	0
*Chrna2*	0	0	0	0	0	0.8	0	0	0	0	0
*Chrna3*	0	2.1	0	0	0	0	0	0	1.6	17.6	0
*Chrna4*	0	2.1	0	0	0	0	0	8.3	0	0	14.6
*Chrna5*	0	2.1	0	0	3.8	0	0	0	0	0	0
*Chrna6*	35.5	2.1	0	0	0	32	31.3	41.7	12.5	0	1.7
*Chrna7*	0	4.2	16.7	0	3.8	0.8	0	0	1.6	11.8	0
*Chrna9*	0	0	0	0	0	0	0	0	0	0	0
*Chrnb1*	0	0	0	0	0	1.6	0	0	3.1	0	0.5
*Chrnb2*	38.7	27.1	58.3	9.1	34.6	35.2	9.4	8.3	6.3	23.5	18.9
*Chrnb3*	0	0	0	0	0	4.8	0	0	7.8	0	0.5
*Chrnb4*	0	0	0	0	0	0.8	0	0	3.1	11.8	0
*Chrnd*	0	0	0	4.5	3.8	0	3.1	0	0	5.9	0
*Chrne*	0	0	0	0	0	0	0	0	0	0	0
*Chrng*	0	0	0	0	3.8	0.8	0	0	0	0	0
*Ngfr*	90.3	70.9	100	18.2	42.3	1.6	31.3	0	42.2	100	46.8

First column indicates gene name. The headers of columns 2–12 indicate the respective class (top row) and subpopulation (second row) of DRG neurons. Numerical values indicate the fraction of positive cells (%) for different neuronal populations. Data was extracted from http://linnarssonlab.org/drg/.

10.1523/JNEUROSCI.1277-17.2017.t1-1 Table 1-1 Genes that are enriched in the TH population and depleted from NF2/3 and PEP2 First column indicates gene name. The headers of columns 2-12 indicate the respective subpopulation of DRG neurons. Numerical values indicate the fraction of positive cells (%) for different neuronal populations. Data was extracted from http://linnarssonlab.org/drg/ (External resource Table 2). Download Table 1-1, DOCX file

10.1523/JNEUROSCI.1277-17.2017.t1-2 Table 1-2 Genes that are depleted from TH population but are expressed in the NF2/3 and PEP2 populations First column indicates gene name. The headers of columns 2-12 indicate the respective subpopulation of DRG neurons. Numerical values indicate the fraction of positive cells (%) for different neuronal populations. Data was extracted from http://linnarssonlab.org/drg/ (External resource Table 2). Download Table 1-2, DOCX file

**Table 2.** Resource table

Reagent	Resource	Identifier
Antibodies (dilution)		
647-IB4 (1:500)	Thermo Fisher Scientific	I32450
Rabbit anti-CGRP (1:1000)	Immunostar	RRID:AB_572217
Chicken anti-GFP (1:1000)	Life Technologies	RRID:AB_2534023
Rabbit anti-GFP (1:1000)	Molecular Probes	RRID:AB_221570
Rabbit anti-Homer1 (1:500)	Synaptic Systems	RRID:AB_2120990
Guinea pig anti-Lmx1b (1:10,000)	Dr. Carmen Birchmeier	Müller et al., 2002
Rat anti-mCherry (1:1000)	Molecular Probes	RRID:AB_2536611
Rabbit anti-NF200 (1:1000)	Sigma-Aldrich	RRID:AB_477272
Rabbit anti-NeuN (1:1000)	Abcam	RRID:AB_10711153
Rabbit anti P2X3 (1:1000)	Abcam	RRID:AB_297006
Sheep anti-PlxnC1 (1:400)	R&D Systems	RRID:AB_2284038
Sheep anti-TH (1:1000)	Millipore	RRID:AB_90755
Goat anti-TrkA (1:400)	R&D Systems	RRID:AB_2283049
Viruses		
AAV1.EF1a.flex.mCherry-2A-SADG	Penn Vector Core (Philadelphia)	Custom production
pAAV1.Ef1a.DIO.oG.WPRE.hGH	Viral Vector Facility (Zurich)	Custom production
SAD.RabiesΔG.eGFP (EnvA)	Produced for this publication	
SAD.RabiesΔG.mCherry (SAD-G)	Produced for this publication	
SAD.RabiesΔG.eGFP (SAD-G)	Produced for this publication/Gene Transfer, Targeting and Therapeutics Core (Salk Institute)	
SAD.RabiesΔG.mCherry (N2C-G)	Produced for this publication	
Mice		
C57BL/6J	Institute of Pharmacology (Zurich)	RRID:IMSR_JAX:000664
129SVJ	Institute of Pharmacology (Zurich)	RRID:IMSR_JAX:000691
Grp::Cre	Mutant Mouse Resource & Research Centers	RRID:MMRRC_037585-UCD
Rosa26^lsl-TVA^	Dieter Saur	Seidler et al., 2008
Rosa26^lsl-tdTomato^	The Jackson Laboratory	RRID:IMSR_JAX:007908
Sns::Cre	Rohini Kuner	Agarwal et al., 2004
Plasmids		
pAAV-Ef1a-DIO-oG-WPRE-hGH	Addgene (RRID:SCR_002037)	74290
pMD.RVG.CVS24-N2c	Addgene (RRID:SCR_002037)	19712

## Discussion

**Figure 8.**
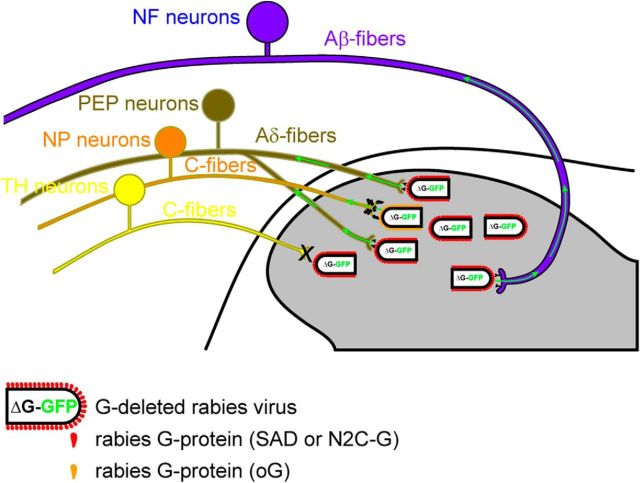
Monosynaptic retrograde tracing is limited to subsets of sensory neurons. The dorsal spinal horn is innervated by myelinated and unmyelinated primary afferent fibers [myelinated: Aβ-fibers and Aδ-fibers from NF and PEP neurons; unmyelinated, C-fibers from NP, PEP, and C-LTMRs (TH)]. G-deleted rabies virus pseudotyped with the rabies glycoproteins SAD19B-G or N2C-G are largely restricted from entering central terminals of NP and TH neurons. Central terminals of NP neurons show some limited susceptibility to infection with G-deleted rabies virus pseudotyped with the optimized rabies glycoprotein (oG).

Here we have shown that two classes of unmyelinated sensory neurons (NP and TH neurons), which account for almost half of the total DRG neuron population, are largely resistant to rabies virus infection from the spinal cord (Fig. 8). These findings have important implications for the interpretation of studies using trans-synaptic rabies virus-based tracing of sensory neurons.

### Limitations of rabies-mediated circuit tracing

Unmyelinated NP and TH sensory nerve fibers serve several important functions in somatosensation. The NP subgroup corresponds mainly to NP unmyelinated fibers that bind IB4 and primarily innervate the skin. In rodents, their activation provokes withdrawal responses or scratching behavior. It is therefore generally accepted that activation of these fibers elicits pain and itch sensations (Basbaum et al., 2009; Braz et al., 2014). TH is a marker for very particular C-type sensory fibers that also innervate the skin, but are activated by low-intensity mechanical stimuli. These fibers are called c-LTMRs (Zotterman, 1939; Li et al., 2011). It is believed that their activation elicits pleasant sensations (Olausson et al., 2010). All of these fiber types terminate either in the superficial layers of the spinal dorsal horn (for those originating in the body trunk and extremities) or in the trigeminal nucleus (in the case of fibers innervating the head). Understanding how these different fibers interact with different types of central neurons is critical for understanding how different sensory modalities are processed. The synaptic contacts underlying these innervation patterns are in most studies assessed with retrograde rabies virus-based tracing experiments. Our results agree with those of several studies that also failed to identify significant input to various dorsal horn neuron populations from NP (IB4+ or P2X3+;Plxnc1+) or TH (TH+) neurons (Bourane et al., 2015a,b; Foster et al., 2015; François et al., 2017). These are unexpected findings, particularly for those dorsal horn neuron populations present in lamina II (Npy+ and Penk+), the termination zone of NP and TH neurons. This finding also contrasts with electrophysiological evidence showing that all morphological classes of lamina II neurons receive input from NP neurons (MrgrpD+ neurons; Wang and Zylka, 2009). One potential reason for the observed resistance to rabies infection could have been neuronal inactivity. Anecdotal clinical results (Willoughby et al., 2005) and unpublished results in the rabies community have suggested that the activity status of neurons might have an impact on infectivity by rabies virus. By injecting formalin immediately after injection of SAD-G-pseudotyped SAD.rabiesΔG-eGFP, we stimulated the activity of NP neurons at the time of infection. Yet, we did not see an increase in the percentage of infected NP neurons. Nevertheless, we detected a significant increase in the overall percentage of infected neurons. Secondary effects of formalin injections are proinflammatory responses, which might alter NF and PEP neurons in a way that increases their susceptibility to rabies infection. In conclusion, we have demonstrated that negative results in retrograde tracing experiments need to be interpreted with greater caution, and additional techniques need to be used to verify the absence of synaptic innervation.

In a recent study addressing the function of spinal Grp neurons, rabies tracing has also been used to identify input to Grp+ spinal interneurons (Sun et al., 2017). Although in that study and in ours the same Grp::Cre transgenic mice have been used, both studies yielded markedly different results. Both studies found >50% of the labeled DRG neurons to be CGRP+;TrkA+. However, while we could not detect any labeling of NP neurons (PlxnC1+;P2X3+) when using SAD-G-pseudotyped rabies, Sun et al. (2017) found that >30% of the labeled DRG neurons were of the NP (IB4+) subtype. These differences are difficult to explain, but differences in age, a previous injury to DRG neurons, or a slightly longer incubation time may provide explanations. A higher susceptibility of NP neurons at early postnatal stages has been suggested by Zhang et al. (2015), who showed that peripheral nociceptors (NP and PEP neurons) can be efficiently infected at P3 (Zhang et al., 2015). Alternatively, a nerve injury potentially caused by the preceding injection of the helper virus (coding for TVA and SAD-G) could result in more neurons being labeled. Solorzano et al. (2015) have indeed demonstrated that Grp is upregulated in DRG neurons upon nerve injury (Solorzano et al., 2015). If nerves were injured during the first injection, TVA may have been expressed in DRG neurons, including neurons of the NP subtype. These neurons would then have been susceptible to direct infection by the EnvA-pseudotyped rabies. Finally, Sun et al. (2017) have used a 7 d incubation time, which was 2 d more than the incubation time we chose for this study. This might allow for the detection of neurons with low levels of retrograde infection.

As previously reported, we also found that DRG neurons were sensitive to infection with the fixed rabies virus strain SAD (Reardon et al., 2016). Prolonged replication and expression of the SAD.RabiesΔG-XFP (≥10 d) led to neurotoxicity and almost complete loss of infected neurons, regardless of whether the modified rabies was pseudotyped with the SAD-G or the N2C-G glycoprotein. It thus seems as though the rapid amplification and expression of the SAD.RabiesΔG-XFP genome is responsible for neurotoxicity. Retrograde tracing experiments using the SAD.RabiesΔG strain in the peripheral nervous system should therefore be limited to ≤7 d after infection.

### Implications for understanding the pathobiology of rabies virus

In the past few decades, significant progress has been made in understanding rabies pathogenesis. The progress has been accomplished by, for example, identifying host proteins that can mediate rabies infection (for review, see Sissoëff et al., 2005; Albertini et al., 2012). Yet, none of the identified proteins are essential for rabies virus infection. Indeed, our analysis of transcriptomic data available from the Linnarsson and Ernfors laboratories (http://linnarssonlab.org/drg/; Usoskin et al., 2015) showed that none of the expression patterns of the identified rabies receptors (nAChR, NCAM, or p75NTR/ Ngfr) could explain the resistance of TH (NP) neurons to rabies infection. Nevertheless, by comparing the transcriptomes of DRG subpopulations susceptible to rabies infection with the transcriptome of the TH population, we identified 321 genes that were lacking in the TH population and 57 that were enriched in the TH population. Pathway analysis of the genes lacking in the TH population indicated significant morphological and structural differences of TH compared with rabies-susceptible DRG neurons. Interestingly, we recently reported that retrograde (axonal) transduction of DRG neurons by AAV also displayed a bias for transduction of NF200+ neurons and against TH and IB4 neurons (Haenraets et al., 2017). It is therefore conceivable that some morphological/structural properties of NP and TH neurons or their central axons impair virus uptake. This might either be due to genes selectively expressed in these populations or absent from them. Future detailed functional analysis of, for example, surface proteins differentially expressed in TH neurons should therefore provide insights into the mechanisms of virus uptake and expression.

### Alternative strategies for the identification of sensory input onto spinal neurons

Rabies-mediated monosynaptic tracing remains a powerful tool for identifying direct input to individual neurons or genetically defined subsets of neurons, and we find that the G-deleted rabies pseudotyped with the recently developed oG glycoprotein leads to a greater infection of connected neurons. However, our results also indicate that if rabies tracing is used to determine peripheral input in adult mice, negative results need to be interpreted with greater caution. Other retrograde tracers, such as pseudorabies or tetanus toxin C-fragment fusion proteins, might be used for verification, but their ability to infect the central terminals of all DRG neuron classes must also be verified first. When using rabies for tracing sensory input, one should complement results obtained with other methods, such as electrophysiology, which has also been used to determine the type of primary afferent input (Wang and Zylka, 2009). In addition, anterograde tracing initiated from distinct DRG subtypes could be used to determine synaptic input from distinct DRG subtypes (Braz et al., 2005). An unbiased approach could be based on the mGRASP (mammalian GFP reconstruction across synaptic partners) system where a pre-mGRASP would be expressed in DRG neurons and a Cre-dependent post-mGRASP in the spinal subpopulation of interest (Kim et al., 2011). Immunohistochemical analyses at the level of GFP-labeled spinal synapses will then provide insights into the type of input provided by primary afferents.
