# Supplementary material for: Identification of Two Classes of Somatosensory Neurons That Display Resistance to Retrograde Infection by Rabies Virus
Source: J Neurosci. 2017 Oct 25;37(43):10358–71. doi: 10.1523/JNEUROSCI.1277-17.2017 (PMC5656993; doi:10.1523/JNEUROSCI.1277-17.2017)
Supplement: Table 1-1 [file zns999170207so1.docx]

|  | **NF1** | **NF2** | **NF3** | **NF4** | **NF5** | **NP1** | **NP2** | **NP3** | **PEP1** | **PEP2** | **TH** |
| --- | --- | --- | --- | --- | --- | --- | --- | --- | --- | --- | --- |
| Tmem45b | 0 | 0.021 | 0 | 0.045 | 0.038 | 0.968 | 0.781 | 1.000 | 0.031 | 0.059 | 0.815 |
| Paqr5 | 0 | 0.021 | 0 | 0 | 0 | 0.736 | 0.438 | 0.083 | 0.016 | 0 | 0.811 |
| Gfra2 | 0.097 | 0.042 | 0 | 0 | 0 | 0.656 | 0.094 | 0 | 0.078 | 0.059 | 0.785 |
| Rasgrp1 | 0 | 0 | 0 | 0 | 0 | 0.528 | 0.250 | 0.167 | 0.016 | 0 | 0.785 |
| Synpr | 0 | 0.063 | 0 | 0 | 0 | 0.864 | 0.688 | 0.083 | 0.047 | 0 | 0.708 |
| Acpp | 0.032 | 0.021 | 0 | 0 | 0 | 0.624 | 0.656 | 0.833 | 0.031 | 0 | 0.695 |
| Th | 0 | 0.021 | 0 | 0 | 0 | 0 | 0 | 0 | 0.016 | 0 | 0.670 |
| Runx1 | 0.065 | 0.021 | 0 | 0.091 | 0 | 0.592 | 0.406 | 0.333 | 0.203 | 0 | 0.631 |
| Rgs8 | 0.032 | 0.042 | 0 | 0 | 0 | 0.288 | 0.313 | 0.333 | 0.016 | 0 | 0.609 |
| Bhlha9 | 0.032 | 0 | 0.083 | 0 | 0.038 | 0.104 | 0.094 | 0.083 | 0.031 | 0 | 0.597 |
| Ccdc68 | 0 | 0.063 | 0 | 0.045 | 0 | 0.568 | 0.031 | 0 | 0 | 0 | 0.562 |
| Ceacam10 | 0 | 0 | 0 | 0 | 0 | 0.008 | 0 | 0 | 0 | 0 | 0.519 |
| Cd34 | 0.032 | 0 | 0.083 | 0 | 0 | 0 | 0.031 | 0.083 | 0.016 | 0.059 | 0.511 |
| Wfdc2 | 0 | 0 | 0 | 0 | 0 | 0 | 0 | 0 | 0 | 0 | 0.502 |
| Slc17a8 | 0 | 0.021 | 0 | 0 | 0 | 0.016 | 0 | 0 | 0.031 | 0.059 | 0.339 |
| Tmem159 | 0.065 | 0.063 | 0 | 0.045 | 0 | 0.200 | 0.094 | 0 | 0.063 | 0.059 | 0.339 |
| Cdh1 | 0.032 | 0.083 | 0 | 0 | 0.038 | 0.472 | 0.094 | 0 | 0.031 | 0 | 0.335 |
| Ldb2 | 0.065 | 0 | 0 | 0 | 0 | 0.352 | 0.438 | 0.750 | 0.344 | 0.059 | 0.330 |
| Cacna1i | 0 | 0 | 0 | 0 | 0 | 0.016 | 0 | 0 | 0 | 0.059 | 0.326 |
| Scn3b | 0.032 | 0.021 | 0 | 0 | 0.038 | 0.040 | 0.281 | 0.333 | 0.094 | 0.059 | 0.326 |
| Arhgap26 | 0.032 | 0 | 0 | 0.091 | 0 | 0.456 | 0.188 | 0.167 | 0.125 | 0 | 0.318 |
| Rnh1 | 0.065 | 0 | 0 | 0.045 | 0 | 0.360 | 0.313 | 0.667 | 0.422 | 0 | 0.309 |
| Litaf | 0.065 | 0.021 | 0 | 0 | 0 | 0.072 | 0.094 | 0.167 | 0.203 | 0 | 0.305 |
| Sorbs2 | 0 | 0.042 | 0.083 | 0 | 0 | 0.032 | 0 | 0.083 | 0.016 | 0 | 0.305 |
| 5730508B09Rik | 0.032 | 0 | 0 | 0.045 | 0 | 0.080 | 0.125 | 0 | 0.047 | 0.059 | 0.296 |
| Aqp1 | 0 | 0.021 | 0 | 0 | 0.038 | 0.376 | 0.094 | 0.083 | 0.609 | 0.059 | 0.292 |
| Arhgap31 | 0 | 0.063 | 0 | 0.091 | 0.154 | 0.056 | 0.031 | 0.083 | 0.047 | 0 | 0.292 |
| Lats2 | 0.065 | 0.083 | 0 | 0 | 0 | 0.200 | 0.219 | 0.083 | 0.156 | 0 | 0.292 |
| Tmem158 | 0 | 0.021 | 0 | 0 | 0 | 0.552 | 0.188 | 0.083 | 0.266 | 0 | 0.283 |
| Bmp15 | 0 | 0 | 0 | 0.045 | 0 | 0.008 | 0.031 | 0 | 0.031 | 0 | 0.279 |
| Fbp2 | 0 | 0.021 | 0 | 0 | 0 | 0.008 | 0 | 0 | 0 | 0 | 0.279 |
| AW551984 | 0 | 0 | 0.083 | 0.045 | 0 | 0 | 0.031 | 0 | 0 | 0.059 | 0.270 |
| Ptrh1 | 0.097 | 0.063 | 0 | 0.091 | 0 | 0.320 | 0.375 | 0.083 | 0.063 | 0 | 0.270 |
| Myt1 | 0 | 0.083 | 0.083 | 0.091 | 0.038 | 0.424 | 0.375 | 0.583 | 0.125 | 0.059 | 0.266 |
| Cdh9 | 0.032 | 0 | 0 | 0.045 | 0 | 0.024 | 0.031 | 0 | 0.031 | 0 | 0.253 |
| Prcp | 0 | 0.083 | 0.083 | 0.091 | 0.077 | 0.096 | 0 | 0.250 | 0.109 | 0 | 0.253 |
| Gpr137b-ps | 0.065 | 0.063 | 0 | 0 | 0 | 0.144 | 0 | 0 | 0.078 | 0.059 | 0.249 |
| Myo10 | 0.065 | 0.063 | 0 | 0 | 0 | 0.040 | 0.031 | 0 | 0 | 0.059 | 0.249 |
| Gramd4 | 0.097 | 0.083 | 0 | 0 | 0 | 0.088 | 0.094 | 0.083 | 0.031 | 0 | 0.245 |
| Pltp | 0 | 0 | 0 | 0 | 0.038 | 0.040 | 0 | 0 | 0 | 0 | 0.240 |
| BC089491 | 0 | 0.021 | 0 | 0.045 | 0 | 0 | 0 | 0 | 0.016 | 0.059 | 0.236 |
| Grhl3 | 0.065 | 0 | 0 | 0.045 | 0 | 0.176 | 0.094 | 0 | 0 | 0.059 | 0.236 |
| Arfgef2 | 0.065 | 0.083 | 0.083 | 0.045 | 0.038 | 0.176 | 0.219 | 0.167 | 0.063 | 0.059 | 0.223 |
| Nxph3 | 0 | 0.021 | 0 | 0.045 | 0.154 | 0.040 | 0.031 | 0 | 0.016 | 0 | 0.223 |
| Pnck | 0.097 | 0.063 | 0.083 | 0.045 | 0 | 0 | 0 | 0 | 0.203 | 0.059 | 0.223 |
| Vim | 0 | 0.083 | 0 | 0.091 | 0.154 | 0.136 | 0.313 | 0.083 | 0.438 | 0 | 0.223 |
| Dapk2 | 0.032 | 0 | 0 | 0 | 0 | 0.232 | 0.031 | 0 | 0 | 0.059 | 0.219 |
| Spinlw1 | 0 | 0 | 0 | 0 | 0.038 | 0.016 | 0 | 0 | 0 | 0.059 | 0.215 |
| Cdkn1b | 0.097 | 0.042 | 0.083 | 0.045 | 0.038 | 0.304 | 0.250 | 0.167 | 0.141 | 0.059 | 0.210 |
| Fam169a | 0.097 | 0.063 | 0.083 | 0.091 | 0.154 | 0.088 | 0.188 | 0.167 | 0.078 | 0.059 | 0.210 |
| Kcnh6 | 0 | 0 | 0.083 | 0 | 0.038 | 0.128 | 0.063 | 0.250 | 0.016 | 0 | 0.210 |
| Ms4a3 | 0 | 0.021 | 0 | 0 | 0 | 0.488 | 0.125 | 0 | 0.031 | 0 | 0.206 |
| Cnp | 0.097 | 0.021 | 0 | 0 | 0 | 0.224 | 0.219 | 0 | 0.234 | 0 | 0.202 |
| Neurl1a | 0.065 | 0.021 | 0.083 | 0.045 | 0 | 0.112 | 0.031 | 0.250 | 0.016 | 0 | 0.202 |
| Nrsn1 | 0 | 0.021 | 0 | 0.045 | 0.154 | 0.016 | 0.344 | 0.333 | 0.328 | 0 | 0.202 |
| Osta | 0 | 0 | 0 | 0 | 0 | 0 | 0 | 0 | 0 | 0 | 0.202 |
| Tram1 | 0.065 | 0.083 | 0.083 | 0 | 0 | 0.112 | 0.063 | 0.167 | 0.047 | 0.059 | 0.202 |

Table 1-1: Genes that are enriched in the TH population and depleted from NF2/3 and PEP2

First column indicates gene name. The headers of columns 2-12 indicate the respective subpopulation of DRG neurons. Numerical values indicate the fraction of positive cells (%) for different neuronal populations. Data was extracted from http://linnarssonlab.org/drg/ (External resource Table 2).
