# Supplementary material for: Identification of Two Classes of Somatosensory Neurons That Display Resistance to Retrograde Infection by Rabies Virus
Source: J Neurosci. 2017 Oct 25;37(43):10358–71. doi: 10.1523/JNEUROSCI.1277-17.2017 (PMC5656993; doi:10.1523/JNEUROSCI.1277-17.2017)
Supplement: Table 1-2 [file zns999170207so2.docx]

|  | **NF1** | **NF2** | **NF3** | **NF4** | **NF5** | **NP1** | **NP2** | **NP3** | **PEP1** | **PEP2** | **TH** |
| --- | --- | --- | --- | --- | --- | --- | --- | --- | --- | --- | --- |
| 1110046J04Rik | 0.258 | 0.417 | 0.333 | 0.227 | 0.154 | 0.056 | 0.094 | 0.083 | 0.078 | 0.294 | 0.047 |
| 1190002N15Rik | 0.032 | 0.188 | 0.333 | 0.364 | 0.346 | 0.032 | 0.156 | 0 | 0.094 | 0.235 | 0.013 |
| 1500001M20Rik | 0.097 | 0.229 | 0.333 | 0.136 | 0.115 | 0.088 | 0.063 | 0.167 | 0.016 | 0.176 | 0.047 |
| 2300005B03Rik | 0.258 | 0.271 | 0.583 | 0 | 0.192 | 0 | 0 | 0 | 0.016 | 0.176 | 0.009 |
| 2810407C02Rik | 0.065 | 0.104 | 0.167 | 0.136 | 0.077 | 0.120 | 0.156 | 0 | 0.094 | 0.118 | 0.026 |
| 4632428C04Rik | 0.194 | 0.188 | 0.167 | 0.091 | 0.115 | 0 | 0 | 0 | 0.078 | 0.118 | 0.009 |
| 4930572J05Rik | 0.258 | 0.146 | 0.417 | 0.045 | 0.115 | 0.024 | 0.063 | 0 | 0.063 | 0.353 | 0.017 |
| 4930578N16Rik | 0.097 | 0.146 | 0.167 | 0.091 | 0.115 | 0.032 | 0.031 | 0 | 0.031 | 0.118 | 0.021 |
| 6330512M04Rik | 0.065 | 0.521 | 1.000 | 0.500 | 0.577 | 0.048 | 0.063 | 0.083 | 0.078 | 0.588 | 0.039 |
| 6430704M03Rik | 0.097 | 0.271 | 0.583 | 0.273 | 0.308 | 0.008 | 0 | 0 | 0.047 | 0.176 | 0.030 |
| 9030425E11Rik | 0.032 | 0.250 | 0.417 | 0.273 | 0.769 | 0.016 | 0 | 0.083 | 0.125 | 0.353 | 0.017 |
| A130010J15Rik | 0.129 | 0.229 | 0.167 | 0.045 | 0.154 | 0.096 | 0.063 | 0.083 | 0.031 | 0.294 | 0.021 |
| Abca8b | 0.194 | 0.208 | 0.167 | 0.136 | 0.115 | 0.008 | 0.031 | 0.167 | 0.031 | 0.118 | 0.043 |
| Abcb1b | 0.065 | 0.146 | 0.250 | 0.045 | 0.154 | 0.008 | 0 | 0 | 0 | 0.235 | 0.013 |
| Ablim2 | 0.258 | 0.292 | 0.500 | 0.318 | 0.577 | 0.008 | 0.094 | 0.167 | 0.016 | 0.118 | 0.021 |
| Acad11 | 0.226 | 0.125 | 0.250 | 0.182 | 0.038 | 0.056 | 0.063 | 0.083 | 0.078 | 0.118 | 0.047 |
| Ache | 0.290 | 0.250 | 0.167 | 0.455 | 0.500 | 0.016 | 0.031 | 0 | 0.266 | 0.118 | 0.047 |
| Acsf3 | 0.161 | 0.167 | 0.167 | 0.227 | 0.538 | 0.056 | 0.125 | 0.083 | 0.047 | 0.176 | 0.047 |
| Adcy3 | 0.129 | 0.146 | 0.417 | 0.136 | 0.154 | 0.008 | 0.031 | 0 | 0.094 | 0.294 | 0.009 |
| Agtrap | 0.226 | 0.292 | 0.500 | 0.091 | 0.385 | 0.232 | 0.375 | 0.583 | 0.078 | 0.118 | 0.026 |
| AI414108 | 0.323 | 0.250 | 0.250 | 0.136 | 0.308 | 0.160 | 0.125 | 0.250 | 0.078 | 0.294 | 0.043 |
| AI464131 | 0.161 | 0.167 | 0.417 | 0.091 | 0.192 | 0.008 | 0.031 | 0 | 0.047 | 0.118 | 0.013 |
| Aifm3 | 0.097 | 0.396 | 0.667 | 0.318 | 0.269 | 0.008 | 0 | 0 | 0.016 | 0.176 | 0.009 |
| Akap1 | 0.161 | 0.188 | 0.250 | 0.091 | 0.231 | 0.120 | 0.063 | 0 | 0.063 | 0.176 | 0.013 |
| Akr1b10 | 0.161 | 0.229 | 0.250 | 0.182 | 0.385 | 0.008 | 0 | 0 | 0 | 0.176 | 0.004 |
| Aldh1l2 | 0 | 0.125 | 0.167 | 0.045 | 0.038 | 0.016 | 0 | 0 | 0 | 0.118 | 0.009 |
| Aldh4a1 | 0.065 | 0.313 | 0.333 | 0.091 | 0.231 | 0.080 | 0.063 | 0.167 | 0.031 | 0.176 | 0.034 |
| Amigo2 | 0 | 0.104 | 0.583 | 0.091 | 0 | 0.016 | 0.031 | 0.167 | 0.141 | 0.176 | 0 |
| Amotl1 | 0.097 | 0.188 | 0.667 | 0.045 | 0.115 | 0.336 | 0.438 | 0.333 | 0.219 | 0.176 | 0.034 |
| Angptl2 | 0 | 0.125 | 0.333 | 0.136 | 0.115 | 0.008 | 0 | 0 | 0 | 0.118 | 0 |
| Ank1 | 0.323 | 0.438 | 0.583 | 0.045 | 0.231 | 0.216 | 0.281 | 0.417 | 0.125 | 0.529 | 0.021 |
| Ankrd29 | 0.452 | 0.479 | 0.750 | 0.182 | 0.385 | 0.088 | 0.063 | 0 | 0.063 | 0.294 | 0.009 |
| Ankrd35 | 0.032 | 0.167 | 0.333 | 0.136 | 0.115 | 0.016 | 0 | 0 | 0.016 | 0.176 | 0.004 |
| Ankrd45 | 0.129 | 0.188 | 0.167 | 0.136 | 0.231 | 0.032 | 0.156 | 0.083 | 0.094 | 0.176 | 0.013 |
| Apeh | 0.516 | 0.271 | 0.333 | 0.409 | 0.346 | 0.208 | 0.188 | 0.250 | 0.156 | 0.294 | 0.021 |
| Arc | 0 | 0.146 | 0.500 | 0.136 | 0.115 | 0 | 0 | 0 | 0 | 0.118 | 0.026 |
| Arhgef19 | 0.194 | 0.167 | 0.167 | 0 | 0.038 | 0.008 | 0 | 0 | 0.016 | 0.176 | 0.009 |
| Arhgef3 | 0.323 | 0.333 | 0.417 | 0.045 | 0.577 | 0.008 | 0 | 0 | 0 | 0.118 | 0.013 |
| Arhgef40 | 0.419 | 0.229 | 0.333 | 0.182 | 0.192 | 0.008 | 0.094 | 0 | 0.031 | 0.353 | 0.039 |
| Arhgef6 | 0.097 | 0.125 | 0.250 | 0.227 | 0.077 | 0.008 | 0.031 | 0 | 0.047 | 0.176 | 0.039 |
| Arid5b | 0.065 | 0.146 | 0.333 | 0.091 | 0.385 | 0 | 0 | 0 | 0.016 | 0.176 | 0.034 |
| Arsg | 0.161 | 0.104 | 0.417 | 0.091 | 0.192 | 0.016 | 0 | 0 | 0.031 | 0.118 | 0 |
| Asah2 | 0.194 | 0.167 | 0.167 | 0.045 | 0.462 | 0.016 | 0.094 | 0 | 0.031 | 0.118 | 0.030 |
| Atp6ap1l | 0.548 | 0.479 | 0.250 | 0.091 | 0.192 | 0.024 | 0.031 | 0 | 0 | 0.176 | 0.009 |
| B230120H23Rik | 0.323 | 0.354 | 0.333 | 0.409 | 0.385 | 0.072 | 0.063 | 0.333 | 0.078 | 0.294 | 0.017 |
| B3galt4 | 0.226 | 0.104 | 0.167 | 0 | 0.077 | 0.064 | 0.094 | 0 | 0 | 0.176 | 0.043 |
| B3galt5 | 0.065 | 0.229 | 0.333 | 0.045 | 0.538 | 0.040 | 0 | 0 | 0.016 | 0.235 | 0.039 |
| B930041F14Rik | 0.355 | 0.438 | 0.417 | 0.273 | 0.385 | 0.008 | 0.156 | 0.083 | 0.094 | 0.412 | 0.026 |
| Baalc | 0.097 | 0.125 | 0.167 | 0 | 0.115 | 0.024 | 0.031 | 0 | 0.094 | 0.294 | 0.030 |
| Bak1 | 0.032 | 0.146 | 0.167 | 0 | 0.192 | 0.008 | 0.063 | 0 | 0.016 | 0.118 | 0.026 |
| Bcat1 | 0.581 | 0.875 | 0.917 | 0.273 | 0.577 | 0.008 | 0.063 | 0 | 0.031 | 0.235 | 0.039 |
| Bdnf | 0.355 | 0.208 | 0.333 | 0 | 0 | 0.312 | 0.156 | 0.250 | 0.297 | 0.412 | 0.009 |
| Bet3l | 0 | 0.125 | 0.750 | 0.182 | 0 | 0.008 | 0.063 | 0.083 | 0.094 | 0.824 | 0.017 |
| Bzrap1 | 0.258 | 0.292 | 0.250 | 0.182 | 0.115 | 0.008 | 0.031 | 0 | 0.016 | 0.176 | 0.017 |
| C1qtnf1 | 0.161 | 0.354 | 0.250 | 0 | 0 | 0.048 | 0.031 | 0.083 | 0.250 | 0.471 | 0.013 |
| C330018D20Rik | 0.097 | 0.208 | 0.333 | 0 | 0.115 | 0.072 | 0 | 0 | 0.016 | 0.176 | 0.039 |
| C530008M17Rik | 0.129 | 0.229 | 0.250 | 0.455 | 0.654 | 0.080 | 0.125 | 0.083 | 0.141 | 0.235 | 0.021 |
| Cad | 0.129 | 0.167 | 0.167 | 0.045 | 0.269 | 0.048 | 0.063 | 0 | 0.016 | 0.176 | 0.034 |
| Cadm2 | 0.097 | 0.167 | 0.167 | 0.136 | 0.231 | 0.208 | 0.094 | 0.167 | 0.094 | 0.294 | 0 |
| Capg | 0.258 | 0.333 | 0.167 | 0.091 | 0.346 | 0.016 | 0.063 | 0 | 0.031 | 0.176 | 0.047 |
| Casc4 | 0.355 | 0.417 | 0.583 | 0.227 | 0.423 | 0.016 | 0.156 | 0.167 | 0.109 | 0.353 | 0.017 |
| Ccbl2 | 0.194 | 0.208 | 0.167 | 0.227 | 0.192 | 0.064 | 0 | 0.083 | 0.047 | 0.353 | 0.017 |
| Ccdc160 | 0.161 | 0.271 | 0.167 | 0.182 | 0.192 | 0.032 | 0.125 | 0.167 | 0.016 | 0.118 | 0.021 |
| Cdk17 | 0.129 | 0.458 | 0.583 | 0.136 | 0.577 | 0.080 | 0.063 | 0 | 0.031 | 0.176 | 0.043 |
| Celsr2 | 0.129 | 0.167 | 0.250 | 0.136 | 0.269 | 0.040 | 0.156 | 0.083 | 0.094 | 0.294 | 0.043 |
| Cenpm | 0.032 | 0.125 | 0.333 | 0.045 | 0 | 0.008 | 0.031 | 0 | 0.016 | 0.118 | 0.004 |
| Cenpv | 0.097 | 0.125 | 0.167 | 0.091 | 0.077 | 0.032 | 0.063 | 0 | 0.031 | 0.118 | 0.043 |
| Cgnl1 | 0.129 | 0.521 | 1.000 | 0.136 | 0.346 | 0.024 | 0.031 | 0 | 0.047 | 0.824 | 0.021 |
| Chml | 0.419 | 0.438 | 0.667 | 0.091 | 0.423 | 0.040 | 0.125 | 0 | 0.063 | 0.294 | 0.026 |
| Chn1 | 0.226 | 0.375 | 0.250 | 0.455 | 0.269 | 0.240 | 0.313 | 0.167 | 0.156 | 0.294 | 0.039 |
| Chrm2 | 0 | 0.104 | 0.417 | 0.182 | 0 | 0.008 | 0 | 0 | 0.016 | 0.176 | 0 |
| Chst1 | 0 | 0.146 | 0.167 | 0 | 0.231 | 0 | 0 | 0 | 0.016 | 0.235 | 0 |
| Chst2 | 0.161 | 0.604 | 0.917 | 0.318 | 0.615 | 0.040 | 0 | 0 | 0.063 | 0.647 | 0.034 |
| Cib1 | 0.226 | 0.125 | 0.333 | 0.136 | 0.115 | 0.104 | 0.188 | 0.167 | 0.094 | 0.118 | 0.017 |
| Clrn1 | 0 | 0.146 | 0.417 | 0.409 | 0.385 | 0.016 | 0 | 0 | 0 | 0.176 | 0.004 |
| Clvs1 | 0.065 | 0.188 | 0.417 | 0.182 | 0.154 | 0.072 | 0.156 | 0.167 | 0.094 | 0.294 | 0.021 |
| Cnksr2 | 0.097 | 0.250 | 0.333 | 0.136 | 0.346 | 0.056 | 0.063 | 0 | 0.031 | 0.176 | 0.030 |
| Cntnap2 | 0 | 0.292 | 0.750 | 1.000 | 0.731 | 0.032 | 0.094 | 0 | 0.078 | 0.412 | 0.026 |
| Col11a1 | 0 | 0.125 | 0.167 | 0 | 0.077 | 0 | 0 | 0 | 0 | 0.294 | 0.034 |
| Cotl1 | 0 | 0.354 | 0.250 | 0.136 | 0.654 | 0.056 | 0 | 0 | 0 | 0.118 | 0.034 |
| Cox15 | 0.161 | 0.146 | 0.250 | 0.045 | 0.269 | 0.064 | 0.031 | 0 | 0.031 | 0.118 | 0.043 |
| Cryab | 0 | 0.646 | 0.750 | 0.136 | 0.077 | 0.032 | 0.031 | 0 | 0.047 | 0.235 | 0.039 |
| Ctdp1 | 0.129 | 0.104 | 0.167 | 0 | 0.038 | 0.072 | 0.031 | 0 | 0.031 | 0.235 | 0.047 |
| Cthrc1 | 0.097 | 0.375 | 0.250 | 0.091 | 0.385 | 0.056 | 0.031 | 0.083 | 0.031 | 0.235 | 0.013 |
| Cyp7b1 | 0.129 | 0.208 | 0.250 | 0.136 | 0.077 | 0.008 | 0 | 0 | 0 | 0.118 | 0.009 |
| D17H6S56E-3 | 0.065 | 0.542 | 0.750 | 0.455 | 0.500 | 0.024 | 0.125 | 0 | 0.094 | 0.529 | 0.026 |
| Dars2 | 0.194 | 0.313 | 0.250 | 0.182 | 0.231 | 0.096 | 0.094 | 0.083 | 0.047 | 0.235 | 0.043 |
| Dbc1 | 0.452 | 0.333 | 0.667 | 0.591 | 0.385 | 0.016 | 0.031 | 0 | 0.125 | 0.471 | 0.004 |
| Dbpht2 | 0.032 | 0.104 | 0.583 | 0.091 | 0.038 | 0 | 0.031 | 0 | 0.031 | 0.412 | 0.009 |
| Ddit4 | 0.097 | 0.146 | 0.250 | 0.091 | 0.154 | 0.032 | 0.063 | 0 | 0.047 | 0.176 | 0.026 |
| Dgkb | 0.032 | 0.438 | 0.417 | 0.136 | 0.385 | 0.008 | 0 | 0 | 0 | 0.118 | 0.004 |
| Dlc1 | 0.161 | 0.146 | 0.167 | 0.136 | 0.269 | 0.096 | 0.063 | 0 | 0.078 | 0.118 | 0.021 |
| Dlg2 | 0.161 | 0.729 | 0.333 | 0.409 | 0.462 | 0.008 | 0 | 0 | 0.031 | 0.118 | 0.030 |
| Dmrtb1 | 0.097 | 0.167 | 0.250 | 0.227 | 0.231 | 0.048 | 0.063 | 0 | 0.031 | 0.118 | 0.030 |
| Dock5 | 0.581 | 0.417 | 1.000 | 0.045 | 0.231 | 0.008 | 0.219 | 0.167 | 0.125 | 0.588 | 0.017 |
| Dtx2 | 0.032 | 0.188 | 0.250 | 0.045 | 0.154 | 0.112 | 0.031 | 0.250 | 0.047 | 0.235 | 0.009 |
| Ebf4 | 0.097 | 0.104 | 0.167 | 0.136 | 0 | 0.008 | 0.031 | 0 | 0.094 | 0.118 | 0.009 |
| Efcab1 | 0.097 | 0.313 | 0.500 | 0.045 | 0 | 0.040 | 0.156 | 0.167 | 0.031 | 0.176 | 0.004 |
| Elfn1 | 0.129 | 0.146 | 0.417 | 0 | 0 | 0 | 0 | 0 | 0.094 | 0.412 | 0.009 |
| Ell2 | 0.419 | 0.375 | 0.667 | 0.091 | 0.385 | 0.064 | 0 | 0.083 | 0.063 | 0.235 | 0.034 |
| Engase | 0.032 | 0.104 | 0.250 | 0 | 0.269 | 0.024 | 0.031 | 0 | 0.047 | 0.235 | 0.030 |
| Entpd6 | 0.097 | 0.250 | 0.333 | 0.227 | 0.385 | 0.096 | 0.031 | 0 | 0.016 | 0.176 | 0.043 |
| Epha6 | 0.129 | 0.208 | 0.250 | 0.091 | 0.077 | 0 | 0 | 0.083 | 0.016 | 0.235 | 0 |
| Epn3 | 0.290 | 0.479 | 0.750 | 0.273 | 0.577 | 0 | 0 | 0 | 0.016 | 0.118 | 0.017 |
| Esr1 | 0.065 | 0.146 | 0.167 | 0.045 | 0.115 | 0.024 | 0.031 | 0 | 0.016 | 0.118 | 0.009 |
| Etaa1 | 0.097 | 0.125 | 0.167 | 0 | 0.154 | 0.048 | 0.031 | 0.083 | 0.063 | 0.176 | 0.039 |
| Etv6 | 0.161 | 0.292 | 0.250 | 0.045 | 0.269 | 0.080 | 0.094 | 0 | 0.078 | 0.176 | 0.043 |
| F2r | 0.290 | 0.271 | 0.667 | 0 | 0.038 | 0 | 0.031 | 0 | 0 | 0.176 | 0.017 |
| Fam155a | 0.194 | 0.438 | 0.500 | 0.182 | 0.577 | 0.008 | 0 | 0.250 | 0.047 | 0.118 | 0.034 |
| Fam19a1 | 0.065 | 0.229 | 0.750 | 0.136 | 0.115 | 0 | 0.063 | 0 | 0.031 | 1.000 | 0.004 |
| Fam35a | 0.129 | 0.125 | 0.417 | 0.045 | 0.192 | 0.056 | 0.031 | 0 | 0.016 | 0.118 | 0.017 |
| Fam86 | 0.161 | 0.104 | 0.167 | 0.091 | 0.154 | 0.080 | 0.031 | 0.250 | 0.078 | 0.118 | 0.047 |
| Fbxl21 | 0.032 | 0.125 | 0.167 | 0 | 0.115 | 0.080 | 0.063 | 0 | 0.031 | 0.118 | 0.004 |
| Fgf18 | 0.226 | 0.271 | 0.167 | 0 | 0.269 | 0 | 0.031 | 0 | 0.078 | 0.353 | 0.004 |
| Fgf9 | 0.032 | 0.479 | 0.500 | 0.091 | 0.577 | 0.024 | 0 | 0.083 | 0.016 | 0.176 | 0.021 |
| Fhit | 0.129 | 0.125 | 0.417 | 0 | 0.154 | 0.096 | 0.063 | 0 | 0.016 | 0.412 | 0.030 |
| Ficd | 0.065 | 0.125 | 0.167 | 0.182 | 0.192 | 0.088 | 0.063 | 0 | 0.031 | 0.294 | 0.043 |
| Folh1 | 0.032 | 0.333 | 0.167 | 0 | 0.154 | 0 | 0 | 0 | 0.016 | 0.235 | 0.009 |
| Fstl5 | 0.419 | 0.458 | 0.500 | 0.455 | 0.731 | 0 | 0.031 | 0 | 0.109 | 0.471 | 0.043 |
| Fxyd7 | 0.516 | 0.604 | 0.917 | 0.500 | 0.192 | 0 | 0 | 0 | 0.578 | 0.824 | 0.004 |
| G0s2 | 0.290 | 0.646 | 0.417 | 0.227 | 0.615 | 0.016 | 0 | 0 | 0.063 | 0.176 | 0.017 |
| G6pd2 | 0.129 | 0.104 | 0.417 | 0 | 0.038 | 0.080 | 0.063 | 0.083 | 0.016 | 0.118 | 0.030 |
| Gadd45b | 0.065 | 0.271 | 0.333 | 0.227 | 0.231 | 0.040 | 0.031 | 0.333 | 0.016 | 0.118 | 0.021 |
| Galnt1 | 0.065 | 0.188 | 0.583 | 0.136 | 0.231 | 0.144 | 0.063 | 0.167 | 0.063 | 0.176 | 0.043 |
| Gas2 | 0.161 | 0.271 | 0.583 | 0.091 | 0.192 | 0.024 | 0 | 0 | 0.016 | 0.176 | 0.026 |
| Gins3 | 0.129 | 0.104 | 0.167 | 0.045 | 0.077 | 0.040 | 0.063 | 0 | 0.031 | 0.118 | 0.039 |
| Gm19461 | 0.419 | 0.500 | 0.417 | 0.182 | 0.462 | 0.024 | 0 | 0.083 | 0.031 | 0.235 | 0.043 |
| Gm20594 | 0.097 | 0.146 | 0.167 | 0.091 | 0.231 | 0.120 | 0.156 | 0.083 | 0.063 | 0.235 | 0.047 |
| Gm6787 | 0.129 | 0.167 | 0.250 | 0.091 | 0.269 | 0.048 | 0.031 | 0 | 0.016 | 0.118 | 0.030 |
| Gm88 | 0.129 | 0.208 | 0.250 | 0.091 | 0.231 | 0.040 | 0 | 0.083 | 0.063 | 0.118 | 0.039 |
| Gm9866 | 0.226 | 0.292 | 0.250 | 0.091 | 0.500 | 0.008 | 0 | 0 | 0.047 | 0.412 | 0.004 |
| Gpr107 | 0.097 | 0.271 | 0.250 | 0.045 | 0.231 | 0.120 | 0.219 | 0.083 | 0.078 | 0.118 | 0.047 |
| Gria2 | 0.032 | 0.417 | 0.250 | 0.318 | 0.577 | 0.080 | 0 | 0.083 | 0.016 | 0.176 | 0.043 |
| Gria4 | 0.194 | 0.500 | 0.333 | 0.136 | 0.538 | 0.032 | 0.219 | 0.167 | 0.125 | 0.353 | 0.047 |
| Grm4 | 0.355 | 0.167 | 0.333 | 0.136 | 0.154 | 0.008 | 0 | 0 | 0 | 0.235 | 0.004 |
| Guf1 | 0.065 | 0.104 | 0.167 | 0 | 0.192 | 0.048 | 0.094 | 0 | 0 | 0.176 | 0.047 |
| Hbq1a | 0.226 | 0.104 | 0.250 | 0 | 0.038 | 0 | 0 | 0 | 0.016 | 0.176 | 0.004 |
| Hexdc | 0.161 | 0.146 | 0.167 | 0.136 | 0.154 | 0.064 | 0.063 | 0.083 | 0.031 | 0.353 | 0.034 |
| Hey1 | 0 | 0.104 | 0.250 | 0.045 | 0.115 | 0.048 | 0.125 | 0.083 | 0.141 | 0.118 | 0.026 |
| Hgf | 0 | 0.188 | 0.167 | 0 | 0.038 | 0.016 | 0 | 0 | 0.016 | 0.118 | 0 |
| Hhatl | 0.419 | 0.583 | 0.583 | 0.591 | 0.846 | 0.024 | 0 | 0 | 0.031 | 0.176 | 0.026 |
| Hist1h2bf | 0.032 | 0.146 | 0.167 | 0 | 0.038 | 0.032 | 0.031 | 0 | 0.016 | 0.176 | 0.043 |
| Hoxd1 | 0.581 | 0.250 | 0.750 | 0.045 | 0.038 | 0.032 | 0.031 | 0.083 | 0.063 | 0.529 | 0.039 |
| Hrasls | 0.194 | 0.313 | 0.167 | 0.091 | 0.577 | 0 | 0 | 0 | 0.047 | 0.176 | 0.034 |
| Hs3st1 | 0.097 | 0.354 | 0.167 | 0 | 0 | 0.008 | 0 | 0 | 0.047 | 0.412 | 0.004 |
| Htr7 | 0.258 | 0.458 | 0.333 | 0.045 | 0.462 | 0.008 | 0 | 0 | 0 | 0.118 | 0.009 |
| Htra1 | 0.452 | 0.625 | 0.833 | 0.500 | 0.615 | 0.032 | 0 | 0 | 0.172 | 0.588 | 0.030 |
| Ifngr1 | 0.290 | 0.167 | 0.167 | 0.136 | 0.077 | 0.040 | 0.156 | 0.083 | 0.125 | 0.118 | 0.030 |
| Igsf21 | 0.065 | 0.208 | 0.250 | 0.273 | 0.192 | 0.008 | 0.063 | 0 | 0.016 | 0.118 | 0 |
| Ikbip | 0.065 | 0.146 | 0.167 | 0.273 | 0.385 | 0.040 | 0.094 | 0.167 | 0.031 | 0.118 | 0.030 |
| Ing2 | 0.032 | 0.125 | 0.167 | 0.045 | 0.077 | 0.040 | 0.063 | 0 | 0.016 | 0.118 | 0.047 |
| Ints7 | 0.097 | 0.125 | 0.167 | 0.091 | 0.154 | 0.048 | 0.063 | 0.083 | 0 | 0.118 | 0.043 |
| Irgm2 | 0.032 | 0.104 | 0.167 | 0 | 0 | 0.216 | 0.156 | 0.083 | 0.078 | 0.353 | 0.034 |
| Itga2b | 0.097 | 0.146 | 0.167 | 0 | 0.154 | 0.016 | 0.063 | 0 | 0.016 | 0.118 | 0.004 |
| Itgb3bp | 0.129 | 0.354 | 0.250 | 0.045 | 0.231 | 0.112 | 0.188 | 0.167 | 0.078 | 0.176 | 0.039 |
| Itpr3 | 0.065 | 0.396 | 0.417 | 0.091 | 0.269 | 0.008 | 0.063 | 0 | 0 | 0.118 | 0.021 |
| Itpripl1 | 0.065 | 0.125 | 0.250 | 0 | 0 | 0.024 | 0.031 | 0 | 0 | 0.235 | 0.043 |
| Jrk | 0.129 | 0.104 | 0.250 | 0.045 | 0.115 | 0.032 | 0 | 0.083 | 0.031 | 0.118 | 0.021 |
| Kcnc3 | 0.065 | 0.417 | 0.417 | 0.318 | 0.731 | 0.040 | 0.031 | 0 | 0.016 | 0.118 | 0.026 |
| Kcnc4 | 0.871 | 0.333 | 0.833 | 0.318 | 0.192 | 0.192 | 0.063 | 0 | 0.250 | 0.353 | 0.026 |
| Kcnq4 | 0 | 0.125 | 0.250 | 0 | 0.038 | 0.008 | 0 | 0.083 | 0 | 0.118 | 0.004 |
| Kcns3 | 0.871 | 0.646 | 0.833 | 0 | 0 | 0.152 | 0.281 | 0 | 0.156 | 0.412 | 0.026 |
| Kifc3 | 0.355 | 0.208 | 0.250 | 0.136 | 0.077 | 0.032 | 0.031 | 0.333 | 0.047 | 0.118 | 0.004 |
| Klhl13 | 0.129 | 0.125 | 0.333 | 0.182 | 0.308 | 0.016 | 0.063 | 0 | 0.047 | 0.118 | 0 |
| Kndc1 | 0.032 | 0.125 | 0.167 | 0.182 | 0.192 | 0 | 0 | 0.083 | 0.063 | 0.235 | 0.017 |
| Lgi3 | 0.516 | 0.917 | 0.917 | 0.773 | 1.000 | 0.064 | 0.031 | 0 | 0.063 | 0.471 | 0.017 |
| Lin7b | 0.323 | 0.667 | 0.667 | 0.500 | 0.654 | 0.088 | 0.063 | 0.083 | 0.063 | 0.588 | 0.047 |
| Lrrc20 | 0.226 | 0.354 | 0.333 | 0.091 | 0.269 | 0 | 0.063 | 0.083 | 0 | 0.118 | 0.047 |
| Lrrc4c | 0 | 0.125 | 0.250 | 0.136 | 0.077 | 0.024 | 0.031 | 0.083 | 0.047 | 0.529 | 0.026 |
| Lrrc61 | 0 | 0.188 | 0.250 | 0 | 0.038 | 0.056 | 0.094 | 0.083 | 0.031 | 0.235 | 0.047 |
| Lrrn2 | 0.290 | 0.583 | 0.667 | 0.227 | 0.192 | 0.008 | 0.063 | 0 | 0.063 | 0.176 | 0 |
| Ltbp3 | 0.161 | 0.396 | 0.833 | 0.364 | 0.731 | 0.088 | 0.344 | 0.083 | 0.016 | 0.176 | 0.030 |
| Maf | 0.129 | 0.604 | 0.833 | 0.045 | 0.385 | 0.024 | 0.125 | 0 | 0.031 | 0.235 | 0.026 |
| Map4k2 | 0.032 | 0.292 | 0.333 | 0.091 | 0.269 | 0.080 | 0.031 | 0 | 0.016 | 0.176 | 0.017 |
| Map6d1 | 0.032 | 0.125 | 0.250 | 0.045 | 0.077 | 0 | 0 | 0 | 0.016 | 0.118 | 0.013 |
| Mapkbp1 | 0 | 0.208 | 0.250 | 0 | 0.154 | 0.032 | 0 | 0 | 0.016 | 0.176 | 0.017 |
| Mchr1 | 0 | 0.104 | 0.167 | 0 | 0.077 | 0 | 0 | 0 | 0 | 0.118 | 0 |
| Mctp1 | 0.226 | 0.458 | 0.167 | 0.136 | 0.077 | 0.040 | 0.250 | 0.417 | 0.016 | 0.353 | 0 |
| Mettl21d | 0.194 | 0.271 | 0.250 | 0.136 | 0.154 | 0.096 | 0.063 | 0 | 0.016 | 0.412 | 0.039 |
| Mgst1 | 0 | 0.146 | 0.583 | 0 | 0.077 | 0 | 0.031 | 0 | 0.031 | 0.471 | 0.021 |
| Mob3b | 0.387 | 0.354 | 0.417 | 0.045 | 0.077 | 0 | 0.094 | 0 | 0.016 | 0.235 | 0.009 |
| mt-Tk | 0.097 | 0.104 | 0.250 | 0.045 | 0.154 | 0.048 | 0.031 | 0 | 0.063 | 0.118 | 0.021 |
| mt-Tw | 0.129 | 0.188 | 0.250 | 0.182 | 0.269 | 0.136 | 0.250 | 0.417 | 0.172 | 0.176 | 0.043 |
| Mudeng | 0.194 | 0.229 | 0.167 | 0.136 | 0.231 | 0.096 | 0.219 | 0.083 | 0.031 | 0.176 | 0.043 |
| Mxra7 | 0.097 | 0.229 | 0.250 | 0.045 | 0.115 | 0.008 | 0.063 | 0 | 0.063 | 0.176 | 0.013 |
| Myh10 | 0.161 | 0.750 | 0.833 | 0.636 | 0.885 | 0.104 | 0.156 | 0 | 0.094 | 0.294 | 0.021 |
| Nebl | 0.129 | 0.125 | 0.250 | 0 | 0.077 | 0.096 | 0.063 | 0 | 0.016 | 0.118 | 0.047 |
| Nefh | 0.806 | 0.958 | 0.917 | 0.727 | 1.000 | 0.008 | 0 | 0 | 0.125 | 0.647 | 0.013 |
| Nefm | 0.742 | 0.979 | 0.917 | 1.000 | 1.000 | 0.136 | 0.250 | 0 | 0.234 | 0.706 | 0.017 |
| Negr1 | 0 | 0.188 | 0.833 | 0.045 | 0.038 | 0.008 | 0 | 0.083 | 0.047 | 0.176 | 0 |
| Nek6 | 0.032 | 0.208 | 0.417 | 0.045 | 0.308 | 0.016 | 0.063 | 0 | 0.063 | 0.176 | 0.009 |
| Nfia | 0.097 | 0.604 | 1.000 | 0.318 | 0.308 | 0.032 | 0.094 | 0 | 0.031 | 0.588 | 0.047 |
| Nhsl1 | 0.290 | 0.208 | 0.250 | 0.091 | 0.192 | 0.024 | 0 | 0.083 | 0.016 | 0.118 | 0.021 |
| Nkain2 | 0.419 | 0.396 | 0.167 | 0.045 | 0.077 | 0.136 | 0.031 | 0.083 | 0.016 | 0.176 | 0.039 |
| Nmral1 | 0.032 | 0.104 | 0.250 | 0.045 | 0 | 0.056 | 0 | 0 | 0.063 | 0.118 | 0.017 |
| Nod1 | 0 | 0.104 | 0.333 | 0.182 | 0.192 | 0.008 | 0.031 | 0 | 0.016 | 0.118 | 0.021 |
| Nrip1 | 0 | 0.125 | 0.417 | 0.182 | 0.231 | 0.176 | 0.250 | 0 | 0.094 | 0.118 | 0.043 |
| Nsmaf | 0.129 | 0.208 | 0.250 | 0.091 | 0.231 | 0.160 | 0.125 | 0.250 | 0.047 | 0.176 | 0.047 |
| Nsun4 | 0.097 | 0.354 | 0.333 | 0.455 | 0.308 | 0.080 | 0.094 | 0.167 | 0.078 | 0.118 | 0.043 |
| Nt5dc3 | 0.161 | 0.354 | 0.500 | 0.136 | 0.038 | 0.096 | 0.156 | 0.083 | 0.141 | 0.353 | 0.047 |
| Olfml2b | 0.226 | 0.438 | 0.417 | 0 | 0.192 | 0.016 | 0.094 | 0 | 0.047 | 0.118 | 0.013 |
| Omg | 0.129 | 0.542 | 0.500 | 0.227 | 0.462 | 0 | 0 | 0 | 0.047 | 0.294 | 0 |
| Osgin2 | 0.065 | 0.188 | 0.333 | 0 | 0.038 | 0.064 | 0.094 | 0 | 0.016 | 0.118 | 0.047 |
| P2rx5 | 0.032 | 0.292 | 0.250 | 0.227 | 0.538 | 0.016 | 0 | 0 | 0.047 | 0.118 | 0.013 |
| P2rx6 | 0.097 | 0.625 | 0.750 | 0.318 | 0.577 | 0.008 | 0 | 0 | 0.016 | 0.176 | 0.034 |
| Paqr8 | 0.097 | 0.250 | 0.417 | 0.091 | 0.154 | 0.128 | 0.094 | 0 | 0.141 | 0.118 | 0.039 |
| Parva | 0.097 | 0.271 | 0.333 | 0.136 | 0.115 | 0.008 | 0.094 | 0 | 0.266 | 0.706 | 0.004 |
| Pcdh17 | 0.258 | 0.417 | 0.750 | 0.091 | 0 | 0.016 | 0.094 | 0.333 | 0.109 | 0.706 | 0.013 |
| Pcdh7 | 0.645 | 0.771 | 0.833 | 0.591 | 0.654 | 0.008 | 0.063 | 0 | 0.063 | 0.118 | 0.034 |
| Pcdh9 | 0.581 | 0.479 | 0.917 | 0.091 | 0.577 | 0.016 | 0 | 0 | 0.172 | 0.294 | 0.034 |
| Pcdhac1 | 0.129 | 0.333 | 0.583 | 0.045 | 0.038 | 0.024 | 0.031 | 0.083 | 0.016 | 0.294 | 0.021 |
| Pcp4l1 | 0.161 | 0.500 | 1.000 | 0 | 0 | 0.008 | 0.094 | 0.083 | 0.141 | 0.647 | 0.009 |
| Pcsk1 | 0.419 | 0.521 | 0.833 | 0.136 | 0.615 | 0.040 | 0.094 | 0 | 0.047 | 0.412 | 0.021 |
| Pcsk2 | 0.742 | 0.896 | 0.750 | 0.864 | 0.615 | 0.760 | 0.750 | 0.417 | 0.250 | 0.353 | 0.047 |
| Pde3b | 0.194 | 0.208 | 0.417 | 0.091 | 0.077 | 0.104 | 0.094 | 0 | 0.094 | 0.294 | 0.039 |
| Pdlim5 | 0 | 0.104 | 0.250 | 0.091 | 0 | 0.264 | 0.375 | 0.333 | 0.109 | 0.176 | 0.013 |
| Pecr | 0.194 | 0.146 | 0.167 | 0.182 | 0.346 | 0.096 | 0.125 | 0.167 | 0.031 | 0.118 | 0.026 |
| Pgr | 0.032 | 0.250 | 0.250 | 0.091 | 0.154 | 0.008 | 0 | 0 | 0.016 | 0.118 | 0.009 |
| Pid1 | 0.226 | 0.292 | 0.500 | 0.045 | 0.231 | 0.008 | 0 | 0 | 0.016 | 0.118 | 0.017 |
| Pigh | 0.032 | 0.208 | 0.167 | 0.045 | 0.346 | 0.072 | 0.031 | 0.083 | 0.031 | 0.118 | 0.030 |
| Pik3r1 | 0.097 | 0.104 | 0.250 | 0.318 | 0.154 | 0.312 | 0.500 | 0.583 | 0.469 | 0.412 | 0.039 |
| Pip5kl1 | 0.065 | 0.250 | 0.583 | 0.091 | 0.154 | 0 | 0 | 0 | 0.016 | 0.118 | 0.017 |
| Pnmal2 | 0.065 | 0.438 | 0.667 | 0.591 | 0.692 | 0.016 | 0.063 | 0 | 0.078 | 0.176 | 0.021 |
| Pou4f3 | 0.129 | 0.125 | 0.333 | 0.091 | 0.115 | 0.008 | 0 | 0 | 0.125 | 0.588 | 0.013 |
| Ppp1r9a | 0 | 0.229 | 0.417 | 0.364 | 0.538 | 0.064 | 0.031 | 0.083 | 0.063 | 0.176 | 0.009 |
| Pprc1 | 0.065 | 0.146 | 0.167 | 0.091 | 0 | 0.072 | 0.094 | 0 | 0.063 | 0.294 | 0.039 |
| Prkcb | 0.097 | 0.188 | 0.417 | 0.318 | 0.538 | 0.008 | 0 | 0 | 0.141 | 0.882 | 0.039 |
| Prmt6 | 0.258 | 0.604 | 0.333 | 0 | 0.269 | 0.088 | 0.031 | 0 | 0.063 | 0.235 | 0.047 |
| Prrt3 | 0.194 | 0.333 | 0.333 | 0.182 | 0.308 | 0.048 | 0 | 0.083 | 0.047 | 0.353 | 0.043 |
| Ptchd2 | 0.097 | 0.271 | 0.167 | 0.045 | 0.115 | 0 | 0.031 | 0 | 0.047 | 0.176 | 0.004 |
| Ptger4 | 0.194 | 0.250 | 0.250 | 0.182 | 0.231 | 0.040 | 0.031 | 0.083 | 0.031 | 0.176 | 0.030 |
| Ptgfrn | 0 | 0.188 | 0.250 | 0.273 | 0.154 | 0.024 | 0 | 0 | 0.016 | 0.235 | 0.021 |
| Ptgr1 | 0.194 | 0.500 | 0.333 | 0.091 | 0.077 | 0.048 | 0.125 | 0.250 | 0.063 | 0.471 | 0.026 |
| Ptprd | 0 | 0.208 | 0.250 | 0.318 | 0.269 | 0.008 | 0.063 | 0 | 0.172 | 0.176 | 0.043 |
| Ptprn | 0.516 | 0.396 | 0.750 | 0.318 | 0.308 | 0.016 | 0.438 | 0.583 | 0.500 | 0.706 | 0.004 |
| Pvrl4 | 0.194 | 0.458 | 0.667 | 0.136 | 0.500 | 0.040 | 0.156 | 0 | 0.047 | 0.353 | 0.039 |
| Qpct | 0 | 0.188 | 0.167 | 0.227 | 0 | 0 | 0.031 | 0 | 0.031 | 0.176 | 0.004 |
| Qpctl | 0.097 | 0.229 | 0.333 | 0 | 0.231 | 0.048 | 0.031 | 0 | 0.031 | 0.235 | 0.039 |
| Rab13 | 0.097 | 0.167 | 0.167 | 0 | 0.192 | 0.008 | 0.031 | 0 | 0.031 | 0.118 | 0.013 |
| Rasgrf2 | 0.097 | 0.438 | 0.833 | 0.273 | 0.308 | 0 | 0 | 0 | 0.016 | 0.353 | 0.004 |
| Rasl10b | 0.258 | 0.500 | 0.750 | 0.273 | 0.577 | 0.008 | 0 | 0 | 0.016 | 0.529 | 0.009 |
| Rcor1 | 0 | 0.104 | 0.167 | 0.045 | 0.231 | 0.024 | 0.031 | 0.083 | 0.031 | 0.176 | 0.047 |
| Recql5 | 0.129 | 0.188 | 0.250 | 0.091 | 0.154 | 0.032 | 0.063 | 0.083 | 0.016 | 0.118 | 0.013 |
| Rell1 | 0.097 | 0.208 | 0.417 | 0 | 0 | 0.016 | 0.031 | 0 | 0.063 | 0.118 | 0 |
| Resp18 | 0.774 | 0.771 | 1.000 | 0.636 | 0.154 | 0.016 | 0.500 | 0.750 | 0.344 | 1.000 | 0.021 |
| Rgs7 | 0.226 | 0.417 | 0.500 | 0.273 | 0.308 | 0.464 | 0.188 | 0.167 | 0.047 | 0.294 | 0.017 |
| Rnf122 | 0.129 | 0.104 | 0.167 | 0 | 0.077 | 0.152 | 0.094 | 0 | 0.016 | 0.176 | 0.013 |
| Rrnad1 | 0.097 | 0.125 | 0.167 | 0.045 | 0.192 | 0.048 | 0.031 | 0.083 | 0.047 | 0.118 | 0.047 |
| Rrs1 | 0.161 | 0.188 | 0.333 | 0 | 0.154 | 0.088 | 0.125 | 0 | 0.063 | 0.294 | 0.047 |
| Rsad1 | 0 | 0.146 | 0.167 | 0 | 0.154 | 0.024 | 0 | 0 | 0 | 0.235 | 0.030 |
| Rwdd2a | 0.097 | 0.167 | 0.250 | 0.318 | 0.115 | 0.064 | 0 | 0.083 | 0.063 | 0.235 | 0.026 |
| Ryr2 | 0.194 | 0.521 | 0.583 | 0.318 | 0.500 | 0.040 | 0.031 | 0 | 0.047 | 0.412 | 0.030 |
| S100a1 | 0.161 | 0.146 | 0.333 | 0.182 | 0.231 | 0.152 | 0.313 | 0.333 | 0.141 | 0.235 | 0.017 |
| S100b | 1.000 | 1.000 | 1.000 | 1.000 | 1.000 | 0.024 | 0.031 | 0 | 0.203 | 1.000 | 0.017 |
| S1pr3 | 0.548 | 0.229 | 0.583 | 0 | 0 | 0.024 | 0 | 0.167 | 0.359 | 0.412 | 0.004 |
| Scx | 0.032 | 0.167 | 0.250 | 0.091 | 0.077 | 0.032 | 0 | 0 | 0 | 0.118 | 0.013 |
| Sema3f | 0 | 0.229 | 0.250 | 0.455 | 0.692 | 0.008 | 0 | 0.250 | 0.125 | 0.118 | 0.009 |
| Serpinb1a | 0 | 0.333 | 0.417 | 0.182 | 0 | 0.040 | 0.063 | 0 | 0.031 | 0.882 | 0.013 |
| Serpinb1b | 0.129 | 0.146 | 0.250 | 0 | 0 | 0.032 | 0.063 | 0.083 | 0.094 | 0.824 | 0 |
| Serpinb1c | 0 | 0.104 | 0.167 | 0 | 0 | 0 | 0.031 | 0 | 0 | 0.235 | 0.004 |
| Sez6 | 0.032 | 0.104 | 0.167 | 0.182 | 0.192 | 0.008 | 0.063 | 0.167 | 0.156 | 0.235 | 0.021 |
| Sfrp1 | 0.032 | 0.146 | 0.583 | 0.182 | 0.038 | 0.008 | 0.031 | 0 | 0.016 | 0.412 | 0.013 |
| Sgcz | 0.161 | 0.333 | 0.333 | 0.136 | 0.115 | 0.040 | 0.094 | 0 | 0.016 | 0.235 | 0.004 |
| Sgsm2 | 0.032 | 0.125 | 0.250 | 0.091 | 0.038 | 0 | 0.094 | 0.083 | 0.188 | 0.176 | 0.026 |
| Sh3kbp1 | 0.097 | 0.292 | 0.250 | 0.136 | 0.154 | 0.008 | 0.094 | 0 | 0.125 | 0.118 | 0.039 |
| Siah2 | 0.290 | 0.104 | 0.250 | 0 | 0.115 | 0.152 | 0.250 | 0.167 | 0.094 | 0.118 | 0.047 |
| Sipa1l1 | 0.065 | 0.229 | 0.417 | 0.182 | 0.308 | 0.016 | 0.063 | 0 | 0.016 | 0.118 | 0.017 |
| Slc16a6 | 0.065 | 0.250 | 0.333 | 0.136 | 0.231 | 0 | 0 | 0 | 0.031 | 0.118 | 0.009 |
| Slc1a1 | 0 | 0.146 | 0.167 | 0.045 | 0.192 | 0 | 0 | 0.083 | 0 | 0.118 | 0 |
| Slc25a29 | 0.161 | 0.208 | 0.417 | 0.045 | 0.038 | 0.040 | 0.063 | 0 | 0.031 | 0.235 | 0.013 |
| Slc25a30 | 0 | 0.125 | 0.250 | 0.045 | 0.115 | 0.016 | 0.094 | 0 | 0.031 | 0.118 | 0.017 |
| Slc29a4 | 0 | 0.188 | 0.500 | 0.409 | 0.231 | 0.136 | 0.219 | 0.083 | 0.141 | 0.471 | 0.004 |
| Slc35b2 | 0.129 | 0.125 | 0.333 | 0.045 | 0.077 | 0.048 | 0.094 | 0 | 0 | 0.176 | 0.039 |
| Slc5a5 | 0.258 | 0.438 | 0.667 | 0.182 | 0.231 | 0.008 | 0 | 0 | 0.094 | 0.412 | 0 |
| Slc7a6 | 0.097 | 0.250 | 0.167 | 0.273 | 0.192 | 0.088 | 0.125 | 0.167 | 0.031 | 0.176 | 0.043 |
| Slc9a3r1 | 0.161 | 0.104 | 0.167 | 0 | 0.038 | 0.032 | 0.031 | 0 | 0.047 | 0.118 | 0.034 |
| Slco3a1 | 0.194 | 0.208 | 0.667 | 0.091 | 0.500 | 0.152 | 0.188 | 0.083 | 0.031 | 0.118 | 0.034 |
| Slitrk1 | 0.065 | 0.479 | 0.750 | 0.455 | 0.462 | 0.032 | 0 | 0 | 0.063 | 0.235 | 0.009 |
| Slmo1 | 0.129 | 0.417 | 0.500 | 0.045 | 0.269 | 0.024 | 0 | 0 | 0.016 | 0.176 | 0.004 |
| Snx24 | 0.065 | 0.208 | 0.167 | 0.091 | 0.269 | 0.016 | 0 | 0 | 0.031 | 0.176 | 0.013 |
| Spsb4 | 0.065 | 0.104 | 0.333 | 0.045 | 0.385 | 0.024 | 0 | 0 | 0 | 0.176 | 0.013 |
| St8sia1 | 0.194 | 0.375 | 0.417 | 0.227 | 0.500 | 0.264 | 0.344 | 0 | 0 | 0.412 | 0.034 |
| Stk32c | 0.484 | 0.313 | 0.500 | 0.045 | 0 | 0.008 | 0.031 | 0 | 0.125 | 0.647 | 0.013 |
| Stxbp5l | 0.129 | 0.292 | 0.333 | 0.455 | 0.538 | 0 | 0 | 0 | 0.094 | 0.294 | 0.004 |
| Supv3l1 | 0.129 | 0.167 | 0.250 | 0.091 | 0.346 | 0.056 | 0.031 | 0 | 0 | 0.118 | 0.047 |
| Sv2b | 0.581 | 0.938 | 0.917 | 0.909 | 0.923 | 0.032 | 0.031 | 0.167 | 0.203 | 0.765 | 0.017 |
| Syt12 | 0.161 | 0.250 | 0.417 | 0.136 | 0.192 | 0.008 | 0 | 0 | 0.016 | 0.235 | 0.017 |
| Syt2 | 0.032 | 0.521 | 0.500 | 0.273 | 0.577 | 0.008 | 0 | 0 | 0.047 | 0.235 | 0.013 |
| Sytl3 | 0 | 0.125 | 0.500 | 0 | 0.038 | 0.048 | 0.094 | 0 | 0.156 | 0.647 | 0 |
| Tacc2 | 0.129 | 0.333 | 0.250 | 0.318 | 0.269 | 0.120 | 0.094 | 0.167 | 0.047 | 0.118 | 0.047 |
| Tbc1d2b | 0.226 | 0.104 | 0.250 | 0.091 | 0.077 | 0.056 | 0.094 | 0 | 0.047 | 0.118 | 0.026 |
| Tbl2 | 0.097 | 0.125 | 0.167 | 0 | 0.077 | 0.080 | 0.063 | 0.083 | 0.016 | 0.118 | 0.013 |
| Tenc1 | 0.097 | 0.146 | 0.500 | 0.045 | 0.038 | 0.024 | 0.063 | 0 | 0.078 | 0.118 | 0.017 |
| Tgfbr1 | 0.065 | 0.208 | 0.250 | 0.091 | 0.077 | 0.024 | 0.063 | 0 | 0.031 | 0.235 | 0.017 |
| Thsd7b | 0.065 | 0.104 | 0.167 | 0.091 | 0.038 | 0.072 | 0.063 | 0 | 0.031 | 0.235 | 0.043 |
| Thy1 | 1.000 | 0.958 | 1.000 | 0.773 | 1.000 | 0 | 0.188 | 0 | 0.203 | 1.000 | 0 |
| Tinf2 | 0.097 | 0.146 | 0.333 | 0 | 0.192 | 0.048 | 0 | 0.083 | 0.016 | 0.235 | 0.047 |
| Tmem143 | 0.097 | 0.146 | 0.583 | 0.136 | 0.192 | 0.064 | 0.031 | 0.167 | 0.063 | 0.235 | 0.017 |
| Tmem229b | 0 | 0.479 | 0.417 | 0.909 | 0.615 | 0.016 | 0 | 0 | 0.109 | 0.529 | 0.026 |
| Tnfrsf12a | 0.129 | 0.271 | 0.417 | 0.227 | 0.346 | 0.088 | 0.094 | 0 | 0.063 | 0.235 | 0.030 |
| Tnfrsf21 | 0.065 | 0.188 | 0.667 | 0.182 | 0.115 | 0.040 | 0.094 | 0.083 | 0.172 | 0.176 | 0.047 |
| Tpcn1 | 0.387 | 0.458 | 0.333 | 0.364 | 0.538 | 0.024 | 0.031 | 0 | 0.047 | 0.176 | 0.047 |
| Traf3ip2 | 0.129 | 0.104 | 0.250 | 0 | 0.077 | 0.056 | 0.094 | 0 | 0.063 | 0.118 | 0.021 |
| Tspan12 | 0.258 | 0.313 | 0.167 | 0.364 | 0.538 | 0.272 | 0.031 | 0.083 | 0.016 | 0.294 | 0.004 |
| Tspyl5 | 0.097 | 0.125 | 0.167 | 0.091 | 0.077 | 0.040 | 0.063 | 0.083 | 0.047 | 0.176 | 0.034 |
| Ttc28 | 0.032 | 0.188 | 0.417 | 0.091 | 0.231 | 0.088 | 0.094 | 0 | 0.063 | 0.118 | 0.004 |
| Ttc39b | 0.065 | 0.396 | 0.167 | 0.273 | 0.462 | 0.104 | 0.063 | 0 | 0.063 | 0.176 | 0.017 |
| Ttf2 | 0.032 | 0.167 | 0.417 | 0.045 | 0.115 | 0.072 | 0.094 | 0 | 0.031 | 0.118 | 0.026 |
| Tyro3 | 0.129 | 0.208 | 0.333 | 0.136 | 0.192 | 0.040 | 0.063 | 0.083 | 0.063 | 0.176 | 0.034 |
| Urb1 | 0.032 | 0.146 | 0.250 | 0 | 0.154 | 0.016 | 0.031 | 0 | 0.031 | 0.118 | 0.026 |
| Vat1l | 0.806 | 0.646 | 1.000 | 0.500 | 0.885 | 0.048 | 0.531 | 0 | 0.203 | 0.588 | 0.030 |
| Vsnl1 | 1.000 | 0.771 | 0.750 | 0.864 | 1.000 | 0 | 0.031 | 0 | 0.063 | 0.588 | 0.013 |
| Vwc2l | 0.129 | 0.333 | 0.250 | 0.045 | 0.154 | 0 | 0.031 | 0 | 0 | 0.235 | 0.017 |
| Wwox | 0.387 | 0.208 | 0.417 | 0.091 | 0.385 | 0.096 | 0.063 | 0.167 | 0.063 | 0.176 | 0.047 |
| Xpo5 | 0.065 | 0.146 | 0.250 | 0 | 0.192 | 0.112 | 0.125 | 0.167 | 0.031 | 0.176 | 0.047 |
| Ypel2 | 0.161 | 0.313 | 0.167 | 0.273 | 0.269 | 0.024 | 0.063 | 0 | 0.031 | 0.118 | 0.043 |
| Ypel4 | 0.161 | 0.271 | 0.167 | 0.227 | 0.154 | 0.112 | 0.063 | 0 | 0.031 | 0.176 | 0.039 |
| Zfp229 | 0.097 | 0.125 | 0.167 | 0.136 | 0 | 0.048 | 0.125 | 0.083 | 0.016 | 0.235 | 0.047 |
| Zfp503 | 0.097 | 0.188 | 0.417 | 0.045 | 0.192 | 0.144 | 0.125 | 0 | 0.016 | 0.294 | 0.009 |
| Zfp612 | 0.065 | 0.271 | 0.417 | 0.182 | 0.308 | 0.088 | 0.063 | 0 | 0.047 | 0.176 | 0.047 |
| Zfp804a | 0 | 0.229 | 0.417 | 0.409 | 0.615 | 0.112 | 0.094 | 0.083 | 0.031 | 0.118 | 0.026 |

Table 1-2: Genes that are depleted from TH population but are expressed in the NF2/3 and PEP2 populations

First column indicates gene name. The headers of columns 2-12 indicate the respective subpopulation of DRG neurons. Numerical values indicate the fraction of positive cells (%) for different neuronal populations. Data was extracted from http://linnarssonlab.org/drg/ (External resource Table 2).
